# Quantitative Metabolomics and Instationary ^13^C-Metabolic Flux Analysis Reveals Impact of Recombinant Protein Production on Trehalose and Energy Metabolism in *Pichia pastoris*

**DOI:** 10.3390/metabo4020281

**Published:** 2014-05-05

**Authors:** Joel Jordà, Hugo Cueto Rojas, Marc Carnicer, Aljoscha Wahl, Pau Ferrer, Joan Albiol

**Affiliations:** 1 Department of Chemical Engineering, Escola d’Enginyeria, Universitat Autònoma de Barcelona, 08193 Bellaterra (Cerdanyola del Vallès), Catalonia, Spain; E-Mails: joel.jorda.murria@gmail.com (J.J.); joan.albiol@uab.cat (J.A.); carnicer@insa-toulouse.fr (M.C.); 2 Department of Biotechnology, Delft University of Technology, 2628 BC Delft, The Netherlands; E-Mail: h.f.cuetorojas@tudelft.nl; 3 Kluyver Centre for Genomics of Industrial Fermentation, Delft University of Technology, 2628 BC Delft, The Netherlands

**Keywords:** *Pichia pastoris*, instationary ^13^C-metabolic flux analysis, recombinant protein production, trehalose, metabolome, GC-MS, LC-MS

## Abstract

*Pichia pastoris* has been recognized as an effective host for recombinant protein production. In this work, we combine metabolomics and instationary ^13^C metabolic flux analysis (INST ^13^C-MFA) using GC-MS and LC-MS/MS to evaluate the potential impact of the production of a *Rhizopus oryzae* lipase (Rol) on *P. pastoris* central carbon metabolism. Higher oxygen uptake and CO_2_ production rates and slightly reduced biomass yield suggest an increased energy demand for the producing strain. This observation is further confirmed by ^13^C-based metabolic flux analysis. In particular, the flux through the methanol oxidation pathway and the TCA cycle was increased in the Rol-producing strain compared to the reference strain. Next to changes in the flux distribution, significant variations in intracellular metabolite concentrations were observed. Most notably, the pools of trehalose, which is related to cellular stress response, and xylose, which is linked to methanol assimilation, were significantly increased in the recombinant strain.

## 1. Introduction

*Pichia pastoris* has become one of the most commonly used expression systems for recombinant protein production during recent years [1,2,3,4]. The overexpression of a recombinant protein may cause a negative influence on cell physiology of *P. pastoris* transformants, particularly in the case of secretory protein production, which is reflected in a reduced methanol consumption capacity and lower specific growth rate [5] or decreased cell viability [6]. Thus, the production and secretion of the protein product seems to influence the intracellular energy balance, transcription and biomass protein formation. Several studies have analyzed the influence on transcription, especially observing changes in the expression of stress related proteins [7,8]. Resina and co-workers [9] reported that Rol overexpression triggered the unfolded protein response (UPR) in *P. pastoris*. To study the impact of the changed cell state on the metabolic properties *in vivo*, advanced approaches including metabolomics and flux analysis are required.

A close link between protein production and energy metabolism is also suggested by the successful strategy to supply the cell with substrate mixtures like mixing methanol with a multi-carbon source such as sorbitol or glycerol [10]. By using such strategies, the production rate of recombinant protein is significantly increased during high cell density cultivations, [11,12,13,14]. Recent quantitative physiological studies have provided new insights on the metabolic burden derived from recombinant protein overexpression in yeast *P. pastoris* and fungi [15,16,17,18]. Most notably, all authors reported that recombinant protein production caused a significant redistribution of carbon fluxes, enabling increased supply of energy resources (NADH, NADPH, ATP) in the producing strains.

The study of the properties of metabolic networks and their regulation *in vivo* is a key field in systems biology and, together with proteomic, transcriptomic or fluxomic studies has become a tool for strain optimization [19,20]. Among the main “-omics” technologies, metabolomics and fluxomics are expected to play a significant role in bridging the phenotype to genotype gap, since it amplifies changes in the proteome and provides a better representation of the phenotype of an organism than other methods [21].

Intracellular fluxes, including cycles, can be determined using ^13^C-based metabolic flux analysis. Especially, the direct measurement of isotopic enrichments of intracellular primary metabolites now allows for short ^13^C labeling experiments (about 1 h) and detailed estimation of fluxes, including fluxes beyond central carbon metabolism [22,23,24].

Recently, Jordà and co-workers [25] applied transient ^13^C metabolic flux analysis to analyze *P. pastoris* growing on glucose/methanol mixtures. The study lead to an extended network topology and a better quantitative characterization of methanol metabolism and related pathways, compared to earlier studies based on ^13^C-labelling of proteinogenic amino acids [18].

In the present study, we quantify the impact of Rol production and secretion on central carbon metabolism using metabolomics and ^13^C transient flux analysis.

## 2. Results and Discussion

### 2.1. Steady-State Chemostat Cultivations

*P. pastoris* cells (X-33/ROL) producing the secreted protein Rol were grown in aerobic, glucose/methanol limited chemostat cultures at a dilution rate of 0.09 h^−1^ (the same conditions as used in our previous study [25]). Under these conditions, biomass, extracellular protein and carbon dioxide were the only products detected, as also observed previously with higher cell densities [18]. Once the steady state was obtained (as reflected in constant macroscopic growth parameters over time), the consumption rates of glucose, methanol and oxygen and the production rates of biomass and carbon dioxide were calculated from measurements of biomass (dry cell weight (DCW)), residual glucose and the concentration of oxygen and carbon dioxide in the off-gas line (Table 1). The experimental data consistency was verified using standard data reconciliation procedures, under the constraint that elemental conservation relations are satisfied [26,27].

**Table 1:** Reconciled specific uptake and production rates of *P. pastoris* growing in chemostat cultures (*D* = 0.09 h^−1^) with glucose/methanol substrate mixture. The calculations and standard deviations are based on triplicate measurements; RQ, Respiratory Quotient. Consistency index h was below 3 for a redundancy of 3 (95% significant level).

Strain	Glucose (mmol/g_DCW_·h)	Methanol (mmol/g_DCW_·h)	OUR (mmol/g_DCW_·h)	CER (mmol/g_DCW_·h)	Biomass (mCmol/g_DCW_·h)	RQ	Lipase Activity (UA/g_DCW_)
X-33 Control	−0.71 ± 0.01	−0.94 ± 0.02	−2.57 ± 0.03	2.03 ± 0.03	3.14 ± 0.04	0.79 ± 0.04	0
X-33/ROL	−0.74 ± 0.03	−1.05 ± 0.05	−2.98 ± 0.04	2.39 ± 0.04	3.08 ± 0.05	0.81 ± 0.06	2417.05 ± 35.4

Based on ^13^C flux analysis results the NAD^+^/NADH and NADP^+^/NADPH balance closed well assuming that all NADH reduction equivalents generated by the proposed network and not consumed within the network, were recycled through the respiratory chain. The ^13^C based oxygen consumption rates represented about 93% of the measured qO_2_ for both strains—no significant imbalance that would suggest major NADPH dehydrogenase or NADPH→NADH transhydrogenase activities.

### 2.2. Intracellular and Extracellular Metabolite Pools

#### 2.2.1. Energy and Redox Cofactors

Protein synthesis and secretion is influenced by adenylate pools [28]. Earlier reports have suggested that the energy charge of *P. pastoris* cell is affected by recombinant protein expression [29]. Hence, it was expected that a lower energy charge (EC) would be found in the Rol-producing strain. Nevertheless, comparing the measurements (Table 2), no significant differences are observed, the energy charge for both X-33/ROL and reference strains is 0.9. In fact, this value is analogous to those calculated for a recombinant *P. pastoris* strain secreting Fab antibody fragment and its control (non producing) strain growing on glucose-limited chemostat cultures, using the same analytical method [30]. Conversely, Plantz and co-workers [29] reported lower EC values for recombinant *P. pastoris* fed-batch cultures, where the EC dropped from 0.75 (just after the transition from the glycerol batch to methanol induction phase) to 0.6 at the end of the methanol feeding phase, mainly due to a steady decrease of ATP and concomitant increase of ADP along this phase. This observation correlated with a decrease in the recombinant protein production rate. Katakura and co-workers [31] also reported a similar effect. Since adenylate pools influence protein synthesis and secretion, these studies support the hypothesis that methanol as a sole carbon source cannot meet the carbon and energy demands to sustain both growth and recombinant protein production for extended periods [29]. In contrast, mixed glucose-methanol steady-state cultures maintained stable Rol production and high energy charge levels. This may explain the improved productivity levels observed in *P. pastoris* fed-batch cultures using mixed methanol/multicarbon source cultures, that is, by using mixed substrates high EC levels may be maintained along all the production phase.

Despite the similarities in the adenylate pools, it is worth noting that GTP produced in the TCA cycle and required for tRNA activation has a slightly higher concentration in the producing strain.

The amount of redox couples NAD^+^/NADH and NADP^+^/NADPH are similar in both strains (Table 2). It is worth noting that the redox state (ratio NAD(P)^+^/NAD(P)H) could not be determined with the applied extraction method. The stability of both NAD as well as NADH is critical during the sampling and especially the extraction procedure. While the reduced pyridine nucleotides (NADH and NADPH) are stable under alkaline conditions, the oxidized pyridine nucleotides (NAD and NADP) are stable under acidic conditions. Additionally, the degradation of both forms is accelerated at elevated temperatures. We cannot therefore exclude degradation or conversion of NAD(H) and NADP(H). Nevertheless, because of the internal standard (^13^C cell extract), losses due to degradation can be compensated and the measured sum of NAD(H) and NADP(H) is quantitative.

**Table 2:** Intracellular concentrations of nucleotides and coenzymes in *P. pastoris* cells growing on glucose/methanol. Concentrations are given in μmol/g_DCW_.

Metabolite	X-33 Control		X-33/ROL	
	Value	Sd	Value	Sd
cAMP	0.01	0.00	0.002	0.00
AMP	0.56	0.37	0.21	0.05
ADP	1.19	0.39	0.86	0.12
ATP	10.10	4.99	8.09	0.79
GMP	0.72	0.11	2.55	2.10
GDP	0.19	0.01	0.17	0.01
GTP	0.99	0.04	1.12	0.03
Acetyl CoA	0.18	0.03	0.21	0.05
FAD	0.73	0.12	1.04	0.06
NAD^+^ + NADH	64.29	13.16	60.07	24.32
NADP^+^ + NADPH	7.47	0.18	8.98	9.14

#### 2.2.2. Central Carbon Metabolism and Storage Metabolites

When comparing the measured intracellular metabolite concentrations of both strains, the majority do follow a comparable profile (Table 3). The absolute values of metabolite concentrations in the Rol-producing strain were generally higher than the control strains ones. However, such differences were only statistically significant for some specific metabolite concentrations. Firstly, the concentrations of all the metabolites of the Pentose Phosphate Pathway (PPP) were statistically significantly higher in the Rol-producing strain (except E4P). Interestingly, the concentration of Xul5P was nearly two-fold higher in the Rol-expressing compared with the control strain, possibly reflecting the increased fraction of methanol being directly oxidized to CO_2_.

**Table 3:** Intra and extracellular metabolite concentrations in *P. pastoris* cells growing on glucose/methanol. Intracellular concentrations (Intra.) of metabolites pools are given in μmol/g_CDW_. Extracellular concentrations (Extra.) of metabolites are given in μmol/L.

Metabolite	X-33 Control				X-33/ROL			
	Intra. (μmol/g_DCW_)		Extra. (μmol/L)		Intra. (μmol/g_DCW_)		Extra. (μmol/L)	
	Value	Sd	Value	Sd	Value	Sd	Value	Sd
Treh	24.50	0.71	1.69	0.06	49.48	4.69	2.72	1.77
Glc6P	14.44	0.52	0.52	0.05	19.04	1.12	0.27	0.04
Citrate	7.17	0.25	n.d	n.d.	6.51	1.60	n.d.	n.d.
Sed7P	5.39	0.20	0.27	0.02	7.96	0.79	0.08	0.12
Fru6P	3.15	0.15	0.17	0.02	4.70	0.39	0.18	0.10
MAL	2.84	2.24	0.09	0.11	4.80	0.23	0.30	0.27
SUCC	1.97	0.15	0.27	0.04	1.29	0.15	0.27	0.26
PG3	1.87	0.10	0.06	0.00	1.79	0.08	0.11	0.07
αKG	1.80	0.19	0.47	0.03	3.09	0.48	3.40	0.40
Pyr	1.47	0.20	43.19	6.28	1.57	0.35	36.02	4.29
Man6P	1.42	0.03	0.14	0.04	1.77	0.09	0.06	0.13
FBP	0.91	0.06	0.21	0.13	0.71	0.13	0.12	0.17
Rib5P	0.88	0.07	0.02	0.00	0.85	0.29	0.08	0.01
Glc	0.78	0.86	35.77	3.99	0.48	0.57	15.86	5.54
FUM	0.77	0.04	0.36	0.02	0.88	0.07	0.73	0.16
Pep	0.76	0.05	0.03	0.05	0.88	0.17	0.18	0.10
DHAP	0.71	0.02	n.d.	n.d.	0.49	0.26	0.02	0.03
Rul5P	0.23	0.03	n.d.	n.d.	0.29	0.03	0.13	0.14
Xul5P	0.16	0.02	n.d.	n.d.	0.34	0.04	0.05	0.03
PG2	0.15	0.06	0.05	0.01	0.22	0.02	0.03	0.03
T6P	0.09	0.01	0.12	0.00	0.26	0.08	0.08	0.07
E4P	0.08	0.00	0.38	0.01	0.19	0.14	0.31	0.23
Isocitrate	0.03	0.03	n.d.	n.d.	0.07	0.02	0.02	0.03
GA3P	0.00	0.00	n.d.	n.d.	0.01	0.01	0.02	0.03

Treh, trehalose; Glc6P, glucose-6-phosphate; Sed7P, sedoheptulose-7-phosphate; Fru6P, fructose-6-phosphate; MAL, malate; SUCC, succinate; PG3, 3-phosphoglycerate; αKG, α-ketoglutarate; Pyr, pyruvate; Man6P, manose-6-phosphate; FBP, fructose-1,6-biphosphate; Rib5P, ribose-5-phosphate; Glc, glucose; FUM, fumarate; Pep, phosphoenolpyruvate; DHAP, dihydroxyacetone phosphate; Rul5P, ribulose-5-phosphate; Xul5P, xylulose-5-phosphate; PG2, 2-phosphoglycerate; T6P, trehalose-6-phosphate; E4P, eritrose-4-phosphate; GA3P, glyceraldehyde-3-phosphate.

Secondly, the trehalose concentration was two-fold higher in the Rol-producing strain. It is well known that yeast (*S. cerevisiae*) is able to increase the trehalose concentration under stress environments [32,33]. Hence, one may speculate that trehalose accumulating to much higher levels in cells producing Rol is in fact reflecting an adaptive response. Resina and co-workers [9] have reported that Rol overexpression triggered the unfolded protein response (UPR) in *P. pastoris*. Moreover, the increase of trehalose could be the result of increased trehalose recycling forming a futile cycle [32], which could contribute to the increased energy demand and lower biomass yield in the X-33/ROL strain.

Glycolytic intermediates seem to be slightly increased in the X-33/ROL strain, which is in agreement with the slightly higher glycolytic flux. Especially, G6P and F6P are increased (32% and 50%, respectively). The increase in F6P could originate from the close link with the non-oxidative pentose phosphate pathway that was in significantly increased concentrations in the producing strain.

The picture for TCA cycle intermediates is not homogeneous. While for malate, isocitrate, fumerate and 2-oxoglutarate higher concentrations were observed, the concentrations for succinate and citrate were lower in the producing strain. It has to be noted that all TCA cycle metabolites are present in the mitochondria and the cytosol. The inhomogeneous trend might indicate that the distribution in the single compartments is changed in the producing compared to the control strain.

For the extracellular metabolite concentrations, no statistically significant differences were generally observed (Table 3). However, there is a general tendency of increased production (or leakage) of central carbon metabolites (except Pyr, FBP, Sed7P and others) and amino acids (except Orn, Asp, see Section 2.3) compared to the control strain. Moreover, the residual extracellular concentration of glucose was significantly lower for the producing strain. This may be reflecting that Rol-producing cells have a slightly higher affinity for glucose transport, specific consumption rate and glycolytic flux compared to the reference strain (Table 1 and Table 3 and [18]).

### 2.3. Intracellular Amino Acid Pools

The free amino acids pools sizes in the Rol-producing *P. pastoris* strain growing on glucose/methanol mixed substrates in this study (Table 4) show a similar overall distribution of amino acids (*i.e.* very high glutamate, glutamine, high Asp and Orn) compared to the reference strain growing on glucose, as previously reported [25,34]. Nevertheless, there are significant differences between the two strains:

(1) The total free amino acid pool in the control strain was 11% lower (288.32 μmol/g_DCW_) compared to the Rol-expressing strain (324.58 μmol/g_DCW_).
(2) In particular, Asp, Orn, Ser, Asn, His, Thr, Pro, Val, Leu, Tyr, Phe pool sizes were statistically significantly higher in the Rol-expressing strain (Table 4), even though the biomass protein production demand of these amino acids was similar for both strains.

**Figure 1:**
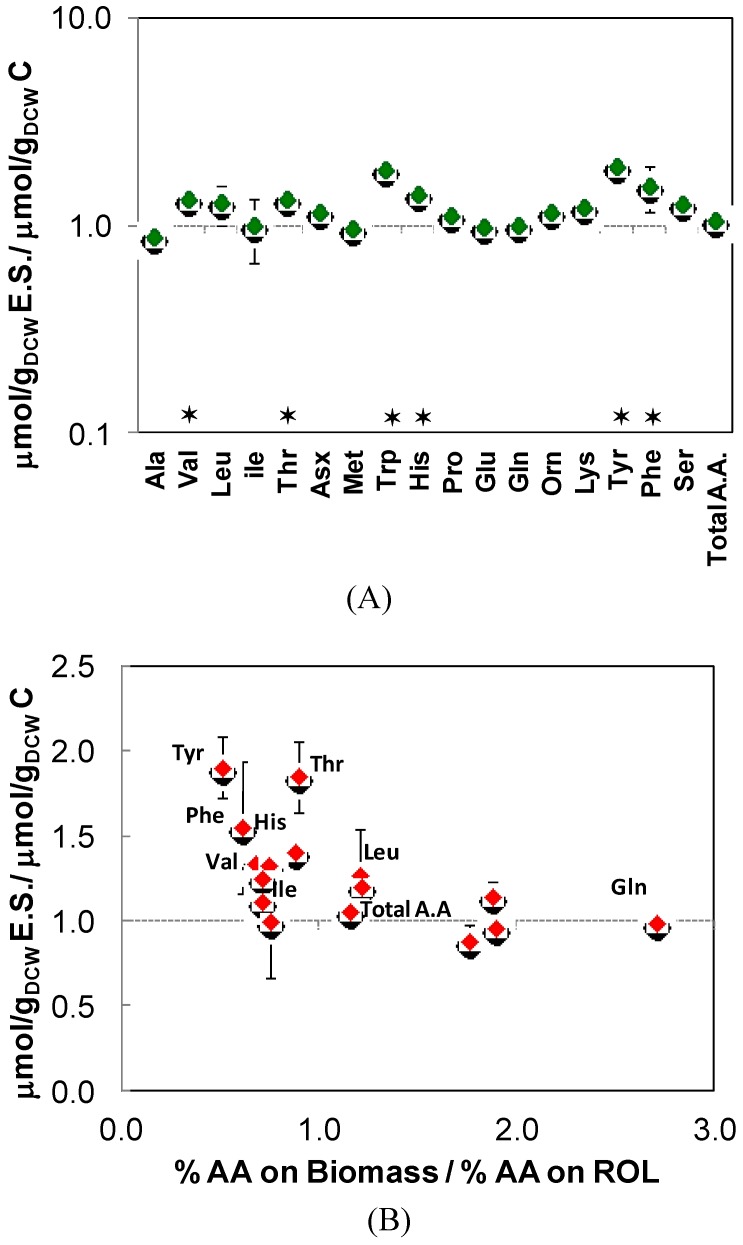
Comparative analysis of amino acid pools. (**A**) Ratio of amino acid pools of the *P. pastoris* Rol-expressing strain (E.S.) and the control strain (C). The error bars are calculated from error propagation. Two-tailed T-test statistical analyses allowed to identify those metabolite ratios that were significantly higher or lower than 1 (marked by a star). (**B**) Intracellular amino acid ratio over the ratio of protein composition (Biomass/Rol).

Hence, there was no apparent correlation between differences in amino acid pool sizes (Figure 1A) and those in cell protein amino acid composition between the two strains. This general trend has also been observed when a Fab fragment is expressed in *P. pastoris* cells grown aerobically on glucose as a sole carbon source [34].

Notably, some of the amino acids showing highest fold change increase in their pools corresponded to those with higher relative abundance in Rol protein with respect to the proteome amino acid composition (Figure 1B). Nevertheless, the addition of the Rol synthesis and secretion rate into the metabolic model did not have an impact on calculated flux distribution (data not shown)—the extracellular Rol protein concentration only reached 0.6–0.8 mg Rol/g_DCW_. This amount is a very small fraction of the total cell protein (less than 1%) and cannot explain the effect observed on cell growth and the metabolic fluxes [18]. Interestingly, this correlation was not observed in another *P. pastoris* strain producing a Fab fragment [34]. Recent isotopic labeling studies on synthesis and secretion of heterologous proteins in *P. pastoris* have reported a very high turnover of the produced protein due to degradation: from the total amount of protein produced intracellular, about 58% was degraded within the cell [35]. Rol overexpression did trigger the UPR, suggesting that there is substantial degradation of misfolded Rol by ER-associated processes. Intracellular Rol degradation could explain higher intracellular levels of those amino acids that are more abundant in the recombinant product. In recombinant strains expressing proteins that cause lower stress levels (less prone to misfolding), less degradation is expected and hence no correlation with the product protein would be observed.

**Table 4:** Intra and extracellular amino acid concentrations in *P. pastoris* cells growing on glucose/methanol. Intracellular concentrations (Intra.) of metabolites pools are given in μmol/g_CDW_. Extracellular concentrations (Extra.) of metabolites are given in μmol/L.

Amino acid	X-33 Control				X-33/ROL			
	Intra. (μmol/g_DCW_)		Extra. (μmol/L)		Intra. (μmol/g_DCW_)		Extra. (μmol/L)	
	Value	Sd	Value	Sd	Value	Sd	Value	Sd
Glu	84.85	2.97	n.d.	n.d.	91.98	4.21	0.03	0.04
Gln	84.97	2.40	0.01	0.00	88.40	3.54	26.05	36.68
Asp	39.56	0.56	0.08	0.00	45.58	3.58	0.03	0.04
Orn	22.59	1.87	1.53	1.10	28.28	1.75	0.16	0.32
Ala	15.01	1.21	1.19	0.22	13.56	0.71	2.77	3.50
Lys	10.22	0.18	0.27	0.22	12.27	0.44	1.15	1.77
Ser	5.94	1.20	n.d	n.d.	9.30	0.38	0.53	0.38
Asn	4.66	0.10	0.11	0.08	9.64	0.39	0.39	0.48
His	4.79	0.15	0.01	0.02	8.13	0.32	0.30	0.43
Gly	1.33	2.44	0.44	0.33	2.35	0.07	2.02	0.87
Thr	2.49	0.21	0.04	0.02	4.24	0.16	0.25	0.04
Pro	2.61	0.07	0.03	0.03	3.85	0.15	0.25	0.16
Val	1.30	0.14	n.d.	n.d.	2.41	0.17	0.24	0.36
Leu	0.69	0.23	0.06	0.04	1.55	0.22	0.39	0.52
Ile	0.33	0.14	0.04	0.02	0.53	0.11	0.22	0.25
Tyr	0.20	0.10	0.03	0.02	1.02	0.04	0.14	0.20
Phe	0.20	0.13	0.02	0.01	0.65	0.09	0.14	0.20
Met	0.48	0.05	n.d.	n.d.	0.53	0.04	0.04	0.07
Trp	0.09	0.03	n.d.	n.d.	0.33	0.02	0.08	0.14

The observation of an apparent adaptation of the amino acid concentration distribution to the recombinant protein composition cannot be solely explained by the increased demand of building blocks for Rol synthesis, or the accumulation of free amino acids as a result of Rol degradation. Moreover, the impact of recombinant protein secretion on the free amino acids pools appears to be at least partially dependent on the specific foreign protein species secreted by the host, even at relatively low expression levels. For instance, this might reflect a complex re-adjustment of the free amino acid pools to compensate for the additional energy demands during recombinant protein overproduction due to intrinsic stresses caused by the unfolded protein response (UPR), as already suggested in [18].

In the same way as for the extracellular levels of the central metabolites, the extracellular amino acids were compared for both strains. As found for the central metabolites, the total concentration of extracellular amino acids in *P. pastoris* control strain chemostat cultivations was lower (total extracellular amino acid pool 3.86 μmol/L) compared with the Rol-expressing strain (9.18 μmol/L). However, when comparing extracellular amino acid pools individually, no statistically significant differences were observed between both strains.

### 2.4. Instationary ^13^C-MFA

The dynamics of most of the measured mass isotopomer distributions for the Rol-expressing strain followed a comparable trend compared to the control strain (Supplementary files 1 and 2) in [25]. There are clear differences observed in the final enrichment of Glc6P, Fru6P and FBP, which are lower in the Rol-expressing strain, as well as in the enrichment dynamics of trehalose (slower in X-33/ROL).

Within a dataset, expected dynamics are observed: The enrichment dynamics of the intermediates of the lower part of the network (TCA cycle) and the storage carbohydrate trehalose were slower compared to glycolysis and PPP metabolites. The glycolytic intermediates reached isotopic steady states after about 10–15 minutes, which is high compared to the turnover time (data not shown) but a commonly observed delay [25,36,37]. The delay can be explained by exchange fluxes with big pools of trehalose and storage polymers such as glycogen.

**Figure 2:**
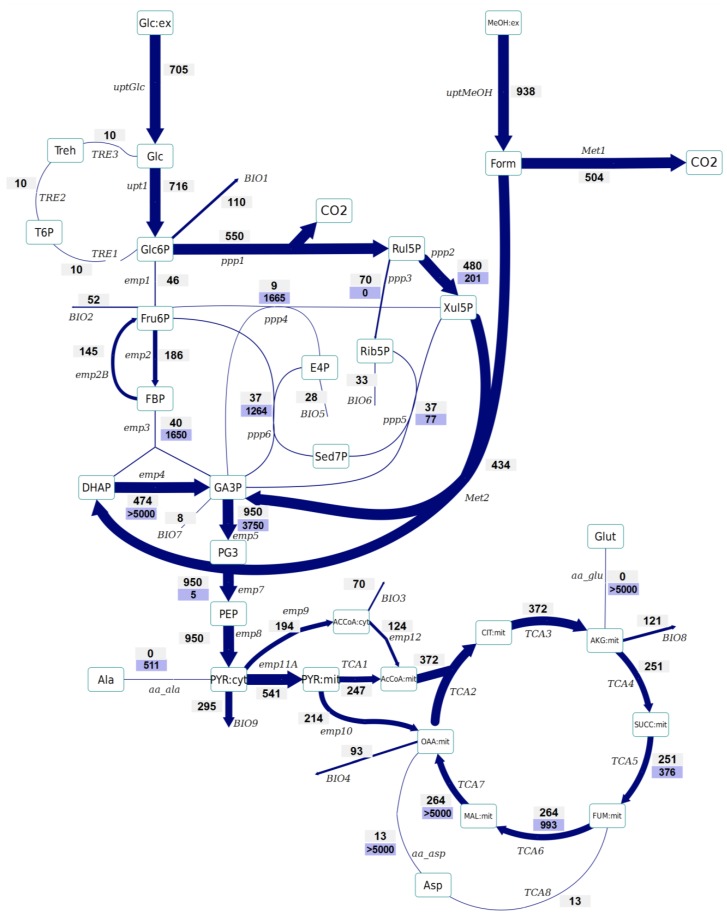
Metabolic flux distributions of the control strain based on INST-^13^C-MFA. The flux values are given in μmol/g_DCW_/h. The width of the arrow represents the flux value. The upper values represent the net flux (in direction of the arrow), whereas the lower values (on blue background) reflect the backward flux (absolute). All the fluxes are also listed in Supplementary File 3.

**Figure 3:**
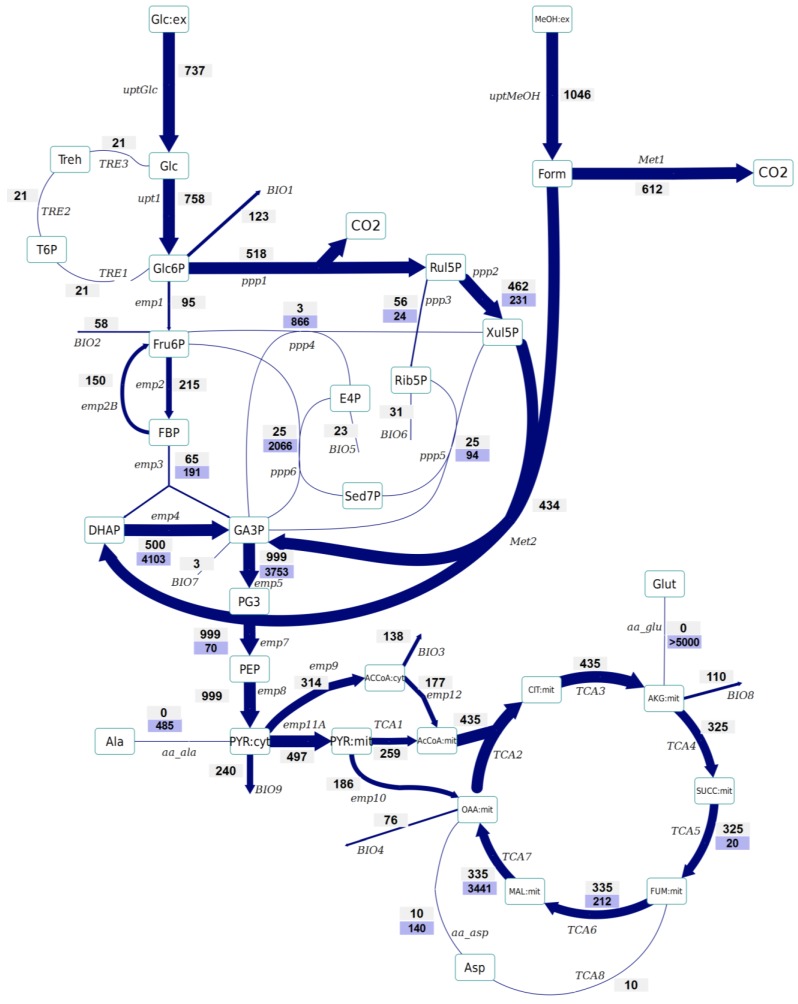
Metabolic flux distributions of the Rol-expressing strain based on INST-^13^C-MFA. The flux values are given in μmol/g_DCW_/h. The width of the arrow represents the flux value. The upper values represent the net flux (in direction of the arrow), whereas the lower values (on blue background) reflect the backward flux (absolute). All the fluxes are also listed in Supplementary File 3.

Based on the labeling enrichment dynamics and measured metabolite concentrations, metabolic fluxes were estimated (Figure 2 and Figure 3).

The metabolic network used contained more detail (fluxes and metabolites) than used in our previous study. Nevertheless, comparing the results for the same reactions, the results of the previous study could be reproduced [18]. These distributions follow the same trend for the different parts of the network.

In particular, the following distinct traits between the Rol-producing and the reference strain could be identified:

- No significant differences have been observed in the amount of Glc6P entering the oxidative branch of the PPP: 77% and 69% of the Glc6P, in the control and Rol-expressing strains, respectively. However, the Rol-producing strain showed a slightly reduced biomass yield. Since the PPP is the main pathway for cytosolic NADPH formation, the flux through the oxidative branch of the PPP is generally directly correlated to the biosynthetic demand for NADPH [38]. The present study further supports the hypothesis of increased NADPH supply through the oxidative branch of the PPP in the Rol-producing strain. To confirm this hypothesis, the NADPH balances were reconstructed taking into account the stoichiometric model and the ^13^C flux estimations (which only balance carbon and labeling). In fact, calculation of NADPH biosynthetic demand for both strains was 0.85 ± 0.03 and 0.97 ± 0.07 mmol/g_DCW_/h for expressing and control strain cells. These values were lower than the total generated NADPH in both cases (1.04 ± 0.16 and 1.10 ± 0.10 mmol/g_DCW_/h, respectively). However, for both strains no statistically significant difference was observed between the generated NADPH and the demanded for biosynthesis. This could reflect the observation that some Crabtree negative yeast appear to have alternative mechanisms involved in the re-oxidation of the NADPH produced in the PPP, e.g. by mitochondrial external alternative dehydrogenases that use NADPH [39]. Alternatively, such effect on the oxidative branch of the PPP could be the indirect consequence of methanol assimilation, which requires Xul5P.
- The Rol-producing strain shows a tendency for a higher fraction of the assimilated methanol being directly oxidized to CO_2_ (60% *vs.* 50%, respectively). This trend can be seen in the enrichment of F6P and DHAP, which is higher (resp. less diluted from unlabelled carbon entering from methanol) in the producing strain. The increased methanol direct oxidation has also been observed using steady-state measurements [18]. The origin could be an increased energy requirement for Rol synthesis and secretion (2 mol NADH per mol of methanol are directly generated in methanol oxidation). Although the increased flux through the methanol oxidative pathway cannot be discriminated statistically, the trend is in agreement with the increased NADH production (increased oxygen uptake) in the Rol producer strain, as well as with our previous study, where such difference was assessed as statistically significant using the ^13^C-NMR based MFA approach [18].
- The flux through the trehalose cycle seems to be altered by Rol production. As it can be seen from Figure 2 and Figure 3, the absolute flux through the trehalose cycle tends to be higher (two-fold) in the Rol-expressing strain. Although such trend is not statistically significant, it is consistent with the statistically significantly higher trehalose concentration observed in this strain, further supporting increased recycling of this molecule building an ATP-futile cycle [32].

Higher trehalose could confer increased protection under stress conditions derived from the UPR [40]. In this context, Schröder and co-workers [41] indentified *TPS2*, encoding for trehalose phosphatase, as one of the genes being induced in an *IRE*- and *HAC1*-independent manner by ER stress with a likely function in alleviating ER stress in yeast.

- The TCA cycle shows a trend towards higher flux for the producing strain. The flux through this part of the network cannot be discriminated statistically, but the trend is in agreement with the increased NADH production (increased oxygen uptake) in the Rol producer, as also observed in our previous study, where such differences were assessed as statistically significant using the ^13^C-NMR based MFA approach [18].
- As previously reported [25], the INST-^13^C approach provides additional insights regarding bidirectional reactions. Notably, the high exchange fluxes for oxaloacetate, malate, and Asp, which indicate amino acid pool buffering and the activity of Malate/Aspartate shuttle [42], are significantly reduced in the Rol-producing strain. In relation to this observation, the turnover time of succinate, fumarate and malate pools is drastically reduced in the producing strain, and the calculated mass action ratio (MAR, based on intracellular concentration measurements) of the fumarase reaction seems to be higher in this strain, although differences are not significant (Supplementary File 4). Also, the Rol-producing strain showed reduced exchange fluxes in some of the reactions of the non-oxidative branch of the PPP in relation to the reference strain, probably reflecting a reduced flux of methanol through its assimilatory pathway.
- The impact of Rol production on the methanol assimilation pathway results in altered behavior of the exchange fluxes in PPP. For instance, the exchange flux of the aldolase reaction was significantly increased in the Rol-producing strain, whereas the exchange flux between DHAP and GA3P was reduced (Figure 2 and Figure 3). As stated above, this trend can be directly observed in the enrichment dynamics of F6P, DHAP, 3PG and 2PG (Supplementary File 9). This may also result in slightly different MAR for the enolase reaction (Supplementary File 4), although this difference was not significant.

## 3. Experimental Section

### 3.1. Strain and Cultivation Conditions

Two recombinant *P. pastoris* X-33 (Invitrogen) derived strains were used in this study. Namely, a control strain harboring pGAPAα (Invitrogen) as mock plasmid [43] and a *Rhizopus oryzae* (Rol)- secreting strain, X-33/pPICZAα-ROL [44], a strain containing a single copy of the ROL expression cassette integrated at the host’s *AOX1* genomic locus.

Duplicate chemostat cultivations were performed in a 2-L bioreactor (Applikon, The Netherlands) with a working volume of 1 L, using the Sartorious Biostat B+ controller. Culture media, growth conditions were the same as described in our previous study [25]. Briefly, cells were grown under carbon-limited conditions at a dilution rate of *D* = 0.09 h^−1^. The aeration rate was set to 1 vvm, controlled by a mass flow controller (5850 Smart Mass Flow Controller, Brooks Instrument). The pO_2_ was always maintained above 40% ensuring fully aerobic conditions. The O_2_ and CO_2_ concentrations in the bioreactor off-gas were measured on-line using a combined paramagnetic/infrared analyzer (NGA 2000, Rosemount, USA). The reported values are based on steady-state off-gas measurements before the switch to ^13^C substrate (^13^CO_2_ has different absorption characteristics). Pressure, pH, stirring speed and temperature were controlled at 1.2 bars, pH 5 (titration with 20% v/v NH_3_), 490 rpm and 25 °C, respectively.

### 3.2. Sampling and Experiment Design

After a minimum of five residence times of continuous cultivation, the feed was switched to the labeled medium. The composition of the labeling feed was the same as described by Jordà and co-workers [25] and the same sampling times were used.

Samples for intracellular metabolite and amino acid concentration measurements were taken one hour before the switch to the labeled feed using a dedicated rapid-sampling setup [45]. Quenching, extraction and further analysis were performed as described earlier [46]. Concentrations were measured from two independent chemostat experiments in triplicate. The labeling enrichment was measured from one wash-in experiment by sampling at 20 time-points following the same procedure as described in [25]. Cell extract samples obtained during the labeling experiment were analyzed by LC/MS and GC/MS [47,48]. The measured mass isotopomers distributions were corrected for natural isotopes of the derivatization reagent (containing carbon as well as non-carbon isotopes) using the MS correction tool [49].

### 3.3. ^13^C-Based Metabolic Flux Analysis (^13^C-MFA)

The metabolic network used in this study for ^13^C-based metabolic flux analyses was the same used in our preceding baseline study [25] (Supplementary File 6). The ^13^C model includes balances for all intracellular metabolites as well as the extracellular substrate. Protein turnover for biomass and product protein was not considered. Nevertheless, large amino acid pools (Glu, Asp, Ala) were added to the model with bidirectional fluxes (synthesis and degradation). Distinct biomass compositions were considered for each strain, based on measurements obtained under analogous growth conditions [18].

The carbon flux distribution of the metabolic reaction network was determined as the best fit (least-squares) of calculated fluxes and mass isotopomers compared to the measured extracellular fluxes and mass isotopomer distributions, weighted by the corresponding experimental variance. The metabolic network model contains 37 metabolite concentrations, required for the dynamic solution, of which a total of 27 were measured (see Supplementary File 3 for details), leaving 10 to be determined from parameter estimation. For the simulation of the labeling enrichments, the instationary approach using the cumomer concept [22] was applied. The solution of the resulting system of ordinary differential equations and the parameter estimation procedure was implemented in gPROMS 3.1 (PSE Limited, London). The parameter estimation routine implemented in gPROMS is based on maximum likelihood (SRQP iterative algorithm). The accuracy of the measurements was described by a constant relative variance: For mass isotopomer measurements a constant relative variance of 2% was used (based on previous experience). For all results presented in this work, a flux fit was considered acceptable when the obtained minimal weighted residual was below the χ^2^ at a 95% confidence level for the corresponding degrees of freedom. The degrees of freedom were calculated as the total number of experimental points (80 mass isotopomer fractions at 20 different times result in a total of 1600 data points) minus the number of parameters (34). As a result of the fitting gPROMS provides the determined parameters together with the standard deviations and 95% confidence intervals (calculated based on error linearized error propagation, see Supplementary File 3).

### 3.4. Analytical Procedures

All the analytical procedures (biomass analysis and quantification of extracellular metabolites) were performed as described earlier [25,46]. The macromolecular and elemental composition, used to derive the biomass effluxes for ^13^C-constrained metabolic flux analysis were obtained from cultures growing under analogous conditions [18]. In all chemostats, the C recovery was above 98%. The experimental data was verified using standard data consistency and reconciliation procedures [26,27,50], under the constraint that the elemental conservation relations are satisfied. For all chemostat cultivations performed, the statistical consistency test was passed at a confidence level of 95%, and consequently there was no indication of gross measurement errors.

The consistency of the measured intracellular metabolite concentrations for the both strains was verified with a method that is based on the second law of thermodynamics [51]. All the calculations were performed using the anNET software developed by Zamboni and co-workers [52] in the same way described in [25] (Supplementary Files 7 and 8).

The feasible ranges of the quantified intracellular metabolites were calculated using the respective experimental mean and standard deviation. The lower and upper limits were defined according to a Student’s *t*-distribution with a confidence interval of 0.85.

## 4. Conclusions

Combined metabolomic and fluxomic analyses has shed light on the impact of heterologous protein secretion on central carbon metabolism of *P. pastoris*, as well as pointing at some adaptive physiologic mechanisms to cope with the metabolic burden caused by foreign protein expression. Remarkably, a clear impact of Rol secretion on trehalose levels was observed, also suggesting an increased flux through the corresponding ATP futile cycle. Increased trehalose levels may contribute to avoid death under UPR stress.

Importantly, the INST-^13^C-MFA methodology yielded coherent results with previous METAFoR-based MFA studies, confirming the impact of Rol overexpression on specific parts of methanol metabolism and TCA cycle, as well as energetic demands. In fact, recent studies suggest that increased ROL copy number could result in increased metabolic burden and stress levels leading to a stronger reduction in biomass and product yields [53]. These series of strains may be a useful model to investigate further the impact of foreign protein overexpression on the energy metabolism of *P. pastoris*. Furthermore, the INST-^13^C-MFA allowed calculating fluxes in a more detailed network—including exchange fluxes in reversible reactions and connected pathways like the trehalose metabolism. Conversely, the use of a larger metabolic network including an increased number of reversible reactions and, therefore, of degrees of freedom, leads to higher uncertainty in the calculated fluxes. Nonetheless, the consistency between independent studies clearly points out that such metabolic flux differences do exist and are not due to experimental variation.

Overall, the combination of two distinct ^13^C-MFA methodologies and quantitative metabolomics illustrates how multi-level omic studies can bring new insights on key modules of cell metabolism in relation to recombinant protein production, which should provide a comprehensive basis for future metabolic engineering of *P. pastoris* for improved recombinant protein production.

## Supplementary Files

Supplementary File 1 Supplementary Materials (ZIP, 2359 KB)
