# Supplementary material for: Quantitative Metabolomics and Instationary 13C-Metabolic Flux Analysis Reveals Impact of Recombinant Protein Production on Trehalose and Energy Metabolism in Pichia pastoris
Source: Metabolites. 2014 May 5;4(2):281–99. doi: 10.3390/metabo4020281 (PMC4101507; doi:10.3390/metabo4020281)
Supplement: Supplementary File 1 — Supplementary Materials (ZIP, 2359 KB) [file metabolites-04-00281-s001.zip › metabolites-04-00281-supplementary/Captions for Supplementary files 1-2-7-8-9.docx]

Captions for Supplementary Files 1, 2, 7, 8 and 9.

Supplementary File 1. Dynamics of mass isotopomers distribution of metabolites of control strain in chemostat cultures after switching to ^13^C-labeled substrates.

Experimental data points are represented as solid circles. Solid lines reflect the simulation with the best flux estimation.

Supplementary File 2. Dynamics of mass isotopomers distribution of metabolites of the Rol-expressing strain in chemostat cultures after switching to ^13^C-labeled substrates. Experimental data points are represented as solid circles. Solid lines reflect the simulation with the best flux estimation.

Supplementary File 7. Transformed Gibbs energy of the cytosolic reactions of the central carbon metabolism of *P. pastoris* growing on glucose/methanol.

(**A**) Transformed Gibbs energies in the control strain. (**B**) Transformed Gibbs energies for the Rol-expressing strain.

Supplementary File 8. Concentrations range of the measured metabolites and the most probable ranges of the non measured ones.

The white bars represent *a priori* considered metabolite ranges, the light grey bars (measured metabolites) and the green bars (unmeasured metabolites) show the corrected values after performing a network-embedded thermodynamic (NET) analysis. In case of detection of a significant metabolite quantification error, the original measurement (red bar) and concentration ranges before and after the NET analysis are shown together.

Supplementary File 9. Comparison of the dynamics of mass isotopomers distribution of metabolites of control *vs* Rol_1 strains growing in chemostat cultures after switching to
^13^C-labeled substrates.

Experimental data points are represented as solid circles (control strain) and empty circles
(Rol-producing strain). Solid and dashed lines reflect the simulation with the best flux estimation for the control and Rol-producing strains, respectively.
