# Supplementary material for: Quantitative Metabolomics and Instationary 13C-Metabolic Flux Analysis Reveals Impact of Recombinant Protein Production on Trehalose and Energy Metabolism in Pichia pastoris
Source: Metabolites. 2014 May 5;4(2):281–99. doi: 10.3390/metabo4020281 (PMC4101507; doi:10.3390/metabo4020281)
Supplement: Supplementary File 1 — Supplementary Materials (ZIP, 2359 KB) [file metabolites-04-00281-s001.zip › metabolites-04-00281-supplementary/Supplementary file 3.docx]

**Supplementary file 3**

Results from the ^13^C flux analysis: Estimated intracellular fluxes under methanol-glucose condition with the calculated standard deviations. The results are in mmol/g_DCW_h.

**Table S3A.** X-33 Control strain flux Distribution.

| **flux** | **value** | **sd** | **flux** | **value** | **sd** |
| --- | --- | --- | --- | --- | --- |
| aa_ala_bwd | 0.511 | 0.044 | feedGlcB_fwd | 0.141 | 0.001 |
| aa_ala_fwd | 0.511 | 0.044 | feedGlcC_fwd | 0.564 | 0.006 |
| aa_asp_bwd | 27.726 | 11.017 | feedMeOHB_fwd | 0.938 | 0.019 |
| aa_asp_fwd | 27.768 | 11.000 | Met1_fwd | 0.504 | 0.071 |
| aa_glu_bwd | 41.163 | 25.000 | Met2_fwd | 0.217 | 0.034 |
| aa_glu_fwd | 41.163 | 25.000 | Met2B_fwd | 0.217 | 0.034 |
| bio1_fwd | 0.110 | 0.005 | Met3_fwd | 0.434 | 0.068 |
| bio2_fwd | 0.052 | 0.002 | Met4_fwd | 0.434 | 0.068 |
| bio3_fwd | 0.070 | 0.034 | ppp1_fwd | 0.550 | 0.098 |
| bio4_fwd | 0.093 | 0.003 | ppp2_bwd | 0.217 | 0.061 |
| bio5_fwd | 0.028 | 0.001 | ppp2_fwd | 0.696 | 0.033 |
| bio6_fwd | 0.033 | 0.002 | ppp3_bwd | 0.000 | 0.064 |
| bio7_fwd | 0.008 | 0.000 | ppp3_fwd | 0.070 | 0.038 |
| bio8_fwd | 0.121 | 0.002 | ppp4_bwd | 1.665 | 0.168 |
| bio9_fwd | 0.295 | 0.008 | ppp4_fwd | 1.674 | 0.160 |
| CO2out1_fwd | 1.904 | 0.104 | ppp5_bwd | 0.077 | 0.140 |
| emp1_bwd | 1.102 | 0.066 | ppp5_fwd | 0.114 | 0.130 |
| emp1_fwd | 1.148 | 0.072 | ppp6_bwd | 1.264 | 0.077 |
| emp10_wd | 0.214 | 0.004 | ppp6_fwd | 1.301 | 0.057 |
| emp11_fwd | 0.001 | 0.012 | TCA1_fwd | 0.247 | 0.055 |
| emp11A_fwd | 0.541 | 0.056 | TCA2_fwd | 0.372 | 0.069 |
| emp11B_fwd | 0.293 | 0.015 | TCA3_fwd | 0.372 | 0.069 |
| emp11C_fwd | 1.087 | 0.072 | TCA4_fwd | 0.125 | 0.035 |
| emp11D_fwd | 1.087 | 0.072 | TCA4B_fwd | 0.125 | 0.035 |
| emp12_fwd | 0.124 | 0.044 | TCA5_bwd | 0.188 | 0.066 |
| emp2_fwd | 0.186 | 0.020 | TCA5_fwd | 0.313 | 0.056 |
| emp2B_fwd | 0.145 | 0.024 | TCA5B_fwd | 0.313 | 0.056 |
| emp3_bwd | 1.650 | 0.621 | TCA5B_bwd | 0.188 | 0.066 |
| emp3_fwd | 1.691 | 0.620 | TCA6_bwd | 0.993 | 1.352 |
| emp4_fwd | 96.980 | 150.000 | TCA6_fwd | 1.257 | 1.200 |
| emp4_bwd | 97.454 | 150.000 | TCA7_bwd | 6.018 | 7.028 |
| emp5_bwd | 3.750 | 4.900 | TCA7_fwd | 6.282 | 7.010 |
| emp5_fwd | 4.700 | 4.900 | TCA8_fwd | 0.013 | 0.021 |
| emp6_bwd | 0.995 | 3.300 | TRE1_fwd | 0.010 | 0.016 |
| emp6_fwd | 1.945 | 3.300 | TRE2_fwd | 0.010 | 0.016 |
| emp7_bwd | 0.005 | 0.096 | TRE3_fwd | 0.010 | 0.016 |
| emp7_fwd | 0.955 | 0.079 | upt1 | 0.716 | 0.001 |
| emp8_fwd | 0.950 | 0.054 | upt2 | 0.938 | 0.019 |
| emp9_fwd | 0.194 | 0.009 | uptGlc | 0.705 | 0.006 |
|  |  |  | uptMeOH | 0.938 | 0.019 |

**Table S3A.** *Cont.*

| **Estimated parameters (μmol/g_DCW_)** | | | | | |
| --- | --- | --- | --- | --- | --- |
| **Metabolites** | **Value** | **Sd** | **Metabolites** | **Value** | **Sd** |
| Form | 2.86 | 1.9 | Metoh_ext_ | 1.414 | 2.3 |
| GAP | 0.0047 | 1.1 | Metoh_int_ | 0.1355 | 1.2 |
| DHA | 0.109 | 0.55 | OAA | 0.1165 | 2.9 |
| ACCoA_mit_ | 0.48 | 5 | CO2 | 0.0338 | 7.3 |

**Table S3B.** X-33 Expressing strain flux distribution.

| **flux** | **value** | **sd** | **flux** | **value** | **sd** |
| --- | --- | --- | --- | --- | --- |
| aa_ala_fwd | 0.485 | 0.031 | feedGlcB_fwd | 0.148 | 0.000 |
| aa_ala_bwd | 0.485 | 0.031 | feedGlcC_fwd | 0.590 | 0.006 |
| aa_asp_bwd | 0.140 | 0.230 | feedMeOHB_fwd | 1.046 | 0.021 |
| aa_asp_fwd | 0.150 | 0.230 | Met1_fwd | 0.612 | 0.261 |
| aa_glu_bwd | 37.777 | 12.000 | Met2_fwd | 0.217 | 0.130 |
| aa_glu_fwd | 37.777 | 12.000 | Met2B_fwd | 0.217 | 0.130 |
| bio1_fwd | 0.123 | 0.006 | Met3_fwd | 0.434 | 0.260 |
| bio2_fwd | 0.058 | 0.003 | Met4_fwd | 0.434 | 0.260 |
| bio3_fwd | 0.138 | 0.002 | ppp1_fwd | 0.518 | 0.160 |
| bio4_fwd | 0.076 | 0.005 | ppp2_bwd | 0.231 | 0.109 |
| bio5_fwd | 0.023 | 0.002 | ppp2_fwd | 0.693 | 0.021 |
| bio6_fwd | 0.031 | 0.001 | ppp3_bwd | 0.024 | 0.103 |
| bio7_fwd | 0.003 | 0.000 | ppp3_fwd | 0.080 | 0.026 |
| bio8_fwd | 0.110 | 0.010 | ppp4_bwd | 0.866 | 0.104 |
| bio9_fwd | 0.240 | 0.017 | ppp4_fwd | 0.869 | 0.087 |
| CO2out1_fwd | 2.277 | 0.378 | ppp5_bwd | 0.094 | 0.133 |
| emp1_bwd | 1.429 | 0.078 | ppp5_fwd | 0.119 | 0.083 |
| emp1_fwd | 1.525 | 0.075 | ppp6_bwd | 2.066 | 0.300 |
| emp10_wd | 0.186 | 0.074 | ppp6_fwd | 2.090 | 0.091 |
| emp11_fwd | 0.001 | 0.015 | TCA1_fwd | 0.259 | 0.136 |
| emp11A_fwd | 0.497 | 0.205 | TCA2_fwd | 0.435 | 0.205 |
| emp11B_fwd | 0.239 | 0.021 | TCA3_fwd | 0.435 | 0.128 |
| emp11C_fwd | 0.987 | 0.130 | TCA4_fwd | 0.163 | 0.090 |
| emp11D_fwd | 0.987 | 0.130 | TCA4B_fwd | 0.163 | 0.090 |
| emp12_fwd | 0.177 | 0.160 | TCA5_bwd | 0.010 | 0.150 |
| emp2_fwd | 0.215 | 0.120 | TCA5_fwd | 0.172 | 0.120 |
| emp2B_fwd | 0.150 | 0.120 | TCA 5B_fwd | 0.172 | 0.120 |
| emp3_bwd | 0.191 | 0.171 | TCA5B_fwd | 0.010 | 0.150 |
| emp3_fwd | 0.257 | 0.019 | TCA6_bwd | 0.212 | 0.637 |
| emp4_bwd | 4.107 | 0.374 | TCA6_fwd | 0.547 | 0.220 |
| emp4_fwd | 4.602 | 0.360 | TCA7_bwd | 3.441 | 2.668 |
| emp5_bwd | 3.757 | 3.201 | TCA7_fwd | 3.777 | 2.700 |
| emp5_fwd | 4.752 | 3.200 | TCA8_fwd | 0.010 | 0.261 |
| emp6_bwd | 7.439 | 4.801 | TRE1_fwd | 0.021 | 0.006 |
| emp6_fwd | 8.434 | 4.800 | TRE2_fwd | 0.021 | 0.006 |
| emp7_bwd | 0.074 | 0.452 | TRE3_fwd | 0.021 | 0.006 |

**Table S3B.** *Cont.*

| **flux** | **value** | **sd** | **flux** | **value** | **sd** |
| --- | --- | --- | --- | --- | --- |
| emp7_fwd | 1.069 | 0.440 | upt1 | 0.758 | 0.000 |
| emp8_fwd | 0.999 | 0.100 | upt2 | 1.046 | 0.021 |
| emp9_fwd | 0.314 | 0.160 | uptGlc | 0.737 | 0.006 |
|  |  |  | uptMeOH | 1.046 | 0.021 |
| **Estimated parameters (μmol/g_DCW_)** | | | | | |
| **Metabolites** | **Value** | **Sd** | **Metabolites** | **Value** | **Sd** |
| Form | 2.51 | 1.3 | Metoh_ext_ | 0.91 | 2.4 |
| GAP | 0.0064 | 0.79 | Metoh_int_ | 0.069 | 0.96 |
| DHA | 0.248 | 0.48 | OAA | 0.13 | 3.3 |
| ACCoA_mit_ | 0.452 | 3.2 | CO_2_ | 0.038 | 8.2 |

© 2014 by the authors; licensee MDPI, Basel, Switzerland. This article is an open access article distributed under the terms and conditions of the Creative Commons Attribution license (http://creativecommons.org/licenses/by/3.0/).
