# Supplementary material for: Quantitative Metabolomics and Instationary 13C-Metabolic Flux Analysis Reveals Impact of Recombinant Protein Production on Trehalose and Energy Metabolism in Pichia pastoris
Source: Metabolites. 2014 May 5;4(2):281–99. doi: 10.3390/metabo4020281 (PMC4101507; doi:10.3390/metabo4020281)
Supplement: Supplementary File 1 — Supplementary Materials (ZIP, 2359 KB) [file metabolites-04-00281-s001.zip › metabolites-04-00281-supplementary/Supplementary file 4 .docx]

**Supplementary file 4**

Measured extra- and intracellular concentrations at steady-state (D = 0.09 h^-1^).

**Table S4A.** Amino acids pools.

| **Amino acids** | **X33_Control_Strain** | | | | **X33_Expresing_Strain** | | | |
| --- | --- | --- | --- | --- | --- | --- | --- | --- |
|  | **Intra. μmol/g_DCW_** | | **Extra. μmol/L** | | **Intra. μmol/g_DCW_** | | **Extra. μmol/L** | |
|  | **Value** | **Sd** | **Value** | **Sd** | **Value** | **Sd** | **Value** | **Sd** |
| Glu | 84.85 | 2.97 | n.d. | n.d. | 91.98 | 4.21 | 0.03 | 0.04 |
| Gln | 84.97 | 2.40 | 0.01 | 0.00 | 88.40 | 3.54 | 26.05 | 36.68 |
| Asp | 39.56 | 0.56 | 0.08 | 0.00 | 45.58 | 3.58 | 0.03 | 0.04 |
| Orn | 22.59 | 1.87 | 1.53 | 1.10 | 28.28 | 1.75 | 0.16 | 0.32 |
| Ala | 15.01 | 1.21 | 1.19 | 0.22 | 13.56 | 0.71 | 2.77 | 3.50 |
| Lys | 10.22 | 0.18 | 0.27 | 0.22 | 12.27 | 0.44 | 1.15 | 1.77 |
| Ser | 5.94 | 1.20 | n.d | n.d. | 9.30 | 0.38 | 0.53 | 0.38 |
| Asn | 4.66 | 0.10 | 0.11 | 0.08 | 9.64 | 0.39 | 0.39 | 0.48 |
| His | 4.79 | 0.15 | 0.01 | 0.02 | 8.13 | 0.32 | 0.30 | 0.43 |
| Gly | 1.33 | 2.44 | 0.44 | 0.33 | 2.35 | 0.07 | 2.02 | 0.87 |
| Thr | 2.49 | 0.21 | 0.04 | 0.02 | 4.24 | 0.16 | 0.25 | 0.04 |
| Pro | 2.61 | 0.07 | 0.03 | 0.03 | 3.85 | 0.15 | 0.25 | 0.16 |
| Val | 1.30 | 0.14 | n.d. | n.d. | 2.41 | 0.17 | 0.24 | 0.36 |
| Leu | 0.69 | 0.23 | 0.06 | 0.04 | 1.55 | 0.22 | 0.39 | 0.52 |
| Ile | 0.33 | 0.14 | 0.04 | 0.02 | 0.53 | 0.11 | 0.22 | 0.25 |
| Tyr | 0.20 | 0.10 | 0.03 | 0.02 | 1.02 | 0.04 | 0.14 | 0.20 |
| Phe | 0.20 | 0.13 | 0.02 | 0.01 | 0.65 | 0.09 | 0.14 | 0.20 |
| Met | 0.48 | 0.05 | n.d. | n.d. | 0.53 | 0.04 | 0.04 | 0.07 |
| Trp | 0.09 | 0.03 | n.d. | n.d. | 0.33 | 0.02 | 0.08 | 0.14 |

**Table S4B.** Metabolites pools.

| **Metabolits** | **X33_Control_Strain** | | | | **X33_Expresing_Strain** | | | |
| --- | --- | --- | --- | --- | --- | --- | --- | --- |
|  | **Intra. μmol/g_DCW_** | | **Extra. μmol/L** | | **Intra. μmol/g_DCW_** | | **Extra. μmol/L** | |
|  | **Value** | **Sd** | **Value** | **Sd** | **Value** | **Sd** | **Value** | **Sd** |
| Treh | 24.50 | 0.71 | 1.69 | 0.06 | 49.48 | 4.69 | 2.72 | 1.77 |
| Glc6P | 14.44 | 0.52 | 0.52 | 0.05 | 19.04 | 1.12 | 0.27 | 0.04 |
| Citrate | 7.17 | 0.25 | n.d | n.d. | 6.51 | 1.60 | n.d. | n.d. |
| Sed7P | 5.39 | 0.20 | 0.27 | 0.02 | 7.96 | 0.79 | 0.08 | 0.12 |
| Fru6P | 3.15 | 0.15 | 0.17 | 0.02 | 4.70 | 0.39 | 0.18 | 0.10 |
| MAL | 2.84 | 2.24 | 0.09 | 0.11 | 4.80 | 0.23 | 0.30 | 0.27 |
| SUCC | 1.97 | 0.15 | 0.27 | 0.04 | 1.29 | 0.15 | 0.27 | 0.26 |
| PG3 | 1.87 | 0.10 | 0.06 | 0.00 | 1.79 | 0.08 | 0.11 | 0.07 |
| αKG | 1.80 | 0.19 | 0.47 | 0.03 | 3.09 | 0.48 | 3.40 | 0.40 |
| Pyr | 1.47 | 0.20 | 43.19 | 6.28 | 1.57 | 0.35 | 36.02 | 4.29 |
| Man6P | 1.42 | 0.03 | 0.14 | 0.04 | 1.77 | 0.09 | 0.06 | 0.13 |
| FBP | 0.91 | 0.06 | 0.21 | 0.13 | 0.71 | 0.13 | 0.12 | 0.17 |
| Rib5P | 0.88 | 0.07 | 0.02 | 0.00 | 0.85 | 0.29 | 0.08 | 0.01 |
| Glu | 0.78 | 0.86 | 35.77 | 3.99 | 0.48 | 0.57 | 15.86 | 5.54 |
| FUM | 0.77 | 0.04 | 0.36 | 0.02 | 0.88 | 0.07 | 0.73 | 0.16 |

**Table S4B.** Metabolites pools.

| **Metabolits** | **X33_Control_Strain** | | | | **X33_Expresing_Strain** | | | |
| --- | --- | --- | --- | --- | --- | --- | --- | --- |
|  | **Intra. μmol/g_DCW_** | | **Extra. μmol/L** | | **Intra. μmol/g_DCW_** | | **Extra. μmol/L** | |
|  | **Value** | **Sd** | **Value** | **Sd** | **Value** | **Sd** | **Value** | **Sd** |
| Pep | 0.76 | 0.05 | 0.03 | 0.05 | 0.88 | 0.17 | 0.18 | 0.10 |
| DHAP | 0.71 | 0.02 | n.d. | n.d. | 0.49 | 0.26 | 0.02 | 0.03 |
| Ribu5P | 0.23 | 0.03 | n.d. | n.d. | 0.29 | 0.03 | 0.13 | 0.14 |
| Xul5P | 0.16 | 0.02 | n.d. | n.d. | 0.34 | 0.04 | 0.05 | 0.03 |
| PG2 | 0.15 | 0.06 | 0.05 | 0.01 | 0.22 | 0.02 | 0.03 | 0.03 |
| T6P | 0.09 | 0.01 | 0.12 | 0.00 | 0.26 | 0.08 | 0.08 | 0.07 |
| E4P | 0.08 | 0.00 | 0.38 | 0.01 | 0.19 | 0.14 | 0.31 | 0.23 |
| IsoCitrate | 0.03 | 0.03 | n.d. | n.d. | 0.07 | 0.02 | 0.02 | 0.03 |
| GA3P | 0.00 | 0.00 | n.d. | n.d. | 0.01 | 0.01 | 0.02 | 0.03 |

**Table S4C.** Comparison of Mass Action Ratios of central carbon metabolism reactions under different cultivations conditions. PGI, Phosphoglucose Isomerase; PMI, Phosphomannose Isomerase; ENO, Enolase; FMH, Fumarase.

| **Enzyme** | **Mass Action Ratios** | **Strain** | | |
| --- | --- | --- | --- | --- |
|  |  | **X-33 Control**  **glucose/methanol** | **X-33/ROL**  **glucose/methanol** | **X-33 Control  glucose ^(a)^** |
| PGI | Fru6P/Glc6P | 0.22 ± 0.03 | 0.25 ± 0.02 | 0.22 ± 0.11 |
| PMI | Man6P/Fru6P | 0.36 ± 0.08 | 0.38 ± 0.06 | 1.27 ± 0.06 |
| ENO | Pep/PG2 | 3.30 ± 0.28 | 3.90 ± 0.98 | 1.67 ± 0.10 |
| FMH | MAL/FUM | 3.70 ± 2.15 | 5.43 ± 1.05 | 5.25 ± 0.38 |

^(a)^ Data taken from (Carnicer *et al.*, 2012 [1]).

References

1. Carnicer, M.; Canelas, A.B.; ten Pierick, A.; Zeng, Z.; van Dam, J.; Albiol, J.; Ferrer, P.; Heijnen, J.J.; van Gulik, W. Development of quantitative metabolomics for Pichia pastoris. *Metabolomics* **2012**, *8*, 284–298.

© 2014 by the authors; licensee MDPI, Basel, Switzerland. This article is an open access article distributed under the terms and conditions of the Creative Commons Attribution license (http://creativecommons.org/licenses/by/3.0/).
