# Supplementary material for: Quantitative Metabolomics and Instationary 13C-Metabolic Flux Analysis Reveals Impact of Recombinant Protein Production on Trehalose and Energy Metabolism in Pichia pastoris
Source: Metabolites. 2014 May 5;4(2):281–99. doi: 10.3390/metabo4020281 (PMC4101507; doi:10.3390/metabo4020281)
Supplement: Supplementary File 1 — Supplementary Materials (ZIP, 2359 KB) [file metabolites-04-00281-s001.zip › metabolites-04-00281-supplementary/Supplementary file 5.docx]

Supplementary file 5

**Table S5A.** Measured mass isotopomers of *P. pastoris* X-33 Control Strain during the wash-in experiment.

|  | **Time (h)** | **0.001** | **0.003** | **0.006** | **0.008** | **0.011** | **0.014** | **0.017** | **0.019** | **0.022** | **0.028** | **0.033** | **0.042** | **0.056** | **0.083** | **0.167** | **0.500** | **1.000** | **2.000** | **4.000** | **6.000** |
| --- | --- | --- | --- | --- | --- | --- | --- | --- | --- | --- | --- | --- | --- | --- | --- | --- | --- | --- | --- | --- | --- |
| FUM | m + 0 | 0.957 | 0.954 | 0.954 | 0.957 | 0.953 | 0.939 | 0.938 | 0.945 | 0.942 | 0.940 | 0.926 | 0.914 | 0.878 | 0.871 | 0.755 | 0.542 | 0.393 | 0.308 | 0.259 | 0.254 |
|  | m + 1 | 0.037 | 0.038 | 0.038 | 0.036 | 0.039 | 0.039 | 0.042 | 0.043 | 0.046 | 0.048 | 0.053 | 0.057 | 0.066 | 0.091 | 0.144 | 0.224 | 0.276 | 0.298 | 0.302 | 0.305 |
|  | m + 2 | 0.005 | 0.007 | 0.006 | 0.007 | 0.008 | 0.020 | 0.020 | 0.011 | 0.011 | 0.011 | 0.018 | 0.023 | 0.042 | 0.023 | 0.057 | 0.129 | 0.187 | 0.223 | 0.244 | 0.244 |
|  | m + 3 | 0.000 | 0.000 | 0.001 | 0.000 | 0.000 | 0.001 | 0.001 | 0.001 | 0.001 | 0.001 | 0.002 | 0.005 | 0.011 | 0.013 | 0.035 | 0.075 | 0.107 | 0.127 | 0.142 | 0.146 |
|  | m + 4 | 0.001 | 0.000 | 0.001 | 0.000 | 0.000 | 0.002 | 0.000 | 0.000 | 0.000 | 0.000 | 0.001 | 0.001 | 0.003 | 0.002 | 0.010 | 0.030 | 0.037 | 0.044 | 0.052 | 0.052 |
| SUCC | m + 0 | 0.963 | 0.963 | 0.963 | 0.963 | 0.964 | 0.962 | 0.960 | 0.961 | 0.959 | 0.958 | 0.953 | 0.947 | 0.936 | 0.910 | 0.830 | 0.627 | 0.459 | 0.351 | 0.292 | 0.271 |
|  | m + 1 | 0.039 | 0.039 | 0.040 | 0.040 | 0.038 | 0.040 | 0.041 | 0.042 | 0.042 | 0.043 | 0.046 | 0.051 | 0.059 | 0.073 | 0.114 | 0.200 | 0.261 | 0.293 | 0.305 | 0.304 |
|  | m + 2 | −0.001 | −0.002 | −0.002 | −0.003 | −0.002 | −0.002 | −0.002 | −0.002 | −0.002 | −0.001 | 0.001 | 0.003 | 0.006 | 0.015 | 0.044 | 0.118 | 0.183 | 0.222 | 0.245 | 0.256 |
|  | m + 3 | 0.000 | 0.000 | −0.001 | 0.000 | −0.001 | 0.000 | 0.000 | −0.001 | 0.000 | 0.000 | −0.001 | −0.001 | −0.001 | 0.001 | 0.009 | 0.042 | 0.077 | 0.104 | 0.123 | 0.129 |
|  | m + 4 | 0.000 | 0.000 | 0.000 | 0.000 | 0.000 | 0.000 | 0.000 | 0.000 | 0.000 | 0.001 | 0.000 | 0.000 | 0.000 | 0.000 | 0.002 | 0.012 | 0.021 | 0.030 | 0.036 | 0.040 |
| MAL | m + 0 | 0.955 | 0.960 | 0.958 | 0.960 | 0.958 | 0.944 | 0.953 | 0.945 | 0.942 | 0.937 | 0.929 | 0.919 | 0.893 | 0.846 | 0.725 | 0.505 | 0.364 | 0.275 | 0.222 | 0.215 |
|  | m + 1 | 0.044 | 0.039 | 0.042 | 0.041 | 0.042 | 0.046 | 0.045 | 0.049 | 0.054 | 0.057 | 0.062 | 0.070 | 0.082 | 0.104 | 0.157 | 0.240 | 0.288 | 0.311 | 0.320 | 0.317 |
|  | m + 2 | 0.000 | −0.002 | −0.003 | −0.003 | −0.003 | −0.002 | −0.002 | −0.001 | 0.000 | 0.001 | 0.003 | 0.004 | 0.011 | 0.028 | 0.065 | 0.140 | 0.192 | 0.231 | 0.248 | 0.252 |
|  | m + 3 | 0.001 | 0.001 | 0.002 | 0.002 | 0.002 | 0.011 | 0.005 | 0.005 | 0.003 | 0.004 | 0.005 | 0.007 | 0.010 | 0.019 | 0.042 | 0.083 | 0.114 | 0.133 | 0.154 | 0.158 |
|  | m + 4 | 0.000 | 0.000 | 0.001 | 0.000 | 0.000 | 0.000 | 0.000 | 0.001 | 0.001 | 0.001 | 0.001 | 0.001 | 0.003 | 0.003 | 0.011 | 0.033 | 0.041 | 0.050 | 0.056 | 0.058 |
| αKG | m + 0 | 0.986 | 0.979 | 0.986 | 0.989 | 0.984 | 0.993 | 0.976 | 0.982 | 0.983 | 0.979 | 0.977 | 0.990 | 0.994 | 0.944 | 0.865 | 0.661 | 0.430 | 0.305 | 0.274 | 0.245 |
|  | m + 1 | 0.006 | 0.009 | 0.008 | 0.001 | 0.005 | 0.006 | 0.015 | 0.005 | 0.003 | 0.004 | 0.010 | 0.007 | 0.012 | 0.050 | 0.098 | 0.175 | 0.247 | 0.276 | 0.264 | 0.280 |
|  | m + 2 | −0.009 | −0.007 | −0.010 | −0.014 | −0.008 | −0.012 | −0.007 | −0.008 | −0.009 | −0.004 | −0.006 | −0.008 | −0.007 | 0.000 | 0.032 | 0.099 | 0.186 | 0.210 | 0.231 | 0.237 |
|  | m + 3 | −0.003 | −0.003 | −0.002 | −0.004 | −0.003 | −0.001 | −0.003 | −0.002 | −0.002 | −0.006 | −0.003 | −0.003 | −0.001 | −0.003 | 0.004 | 0.032 | 0.083 | 0.120 | 0.146 | 0.150 |
|  | m + 4 | 0.019 | 0.019 | 0.015 | 0.025 | 0.020 | 0.011 | 0.016 | 0.021 | 0.021 | 0.022 | 0.019 | 0.012 | 0.001 | 0.025 | 0.016 | 0.021 | 0.032 | 0.055 | 0.069 | 0.066 |
|  | m + 5 | 0.002 | 0.003 | 0.003 | 0.003 | 0.003 | 0.003 | 0.003 | 0.002 | 0.003 | 0.005 | 0.003 | 0.002 | 0.002 | 0.004 | 0.004 | 0.012 | 0.012 | 0.023 | 0.016 | 0.021 |
| PG2 | m + 0 | 0.952 | 0.926 | 0.860 | 0.812 | 0.738 | 0.709 | 0.671 | 0.626 | 0.615 | 0.573 | 0.524 | 0.513 | 0.495 | 0.457 | 0.412 | 0.408 | 0.421 | 0.437 | 0.452 | 0.428 |
|  | m + 1 | 0.037 | 0.074 | 0.113 | 0.159 | 0.202 | 0.226 | 0.255 | 0.271 | 0.261 | 0.271 | 0.286 | 0.279 | 0.265 | 0.261 | 0.242 | 0.240 | 0.235 | 0.244 | 0.228 | 0.241 |
|  | m + 2 | 0.006 | −0.011 | 0.020 | 0.017 | 0.033 | 0.039 | 0.027 | 0.050 | 0.064 | 0.077 | 0.106 | 0.120 | 0.131 | 0.144 | 0.186 | 0.174 | 0.176 | 0.166 | 0.161 | 0.161 |
|  | m + 3 | 0.005 | 0.011 | 0.007 | 0.011 | 0.027 | 0.027 | 0.048 | 0.053 | 0.060 | 0.080 | 0.084 | 0.089 | 0.110 | 0.138 | 0.160 | 0.177 | 0.168 | 0.153 | 0.159 | 0.170 |

**Table S5A.** *Cont.*

|  | **Time (h)** | **0.001** | **0.003** | **0.006** | **0.008** | **0.011** | **0.014** | **0.017** | **0.019** | **0.022** | **0.028** | **0.033** | **0.042** | **0.056** | **0.083** | **0.167** | **0.500** | **1.000** | **2.000** | **4.000** | **6.000** |
| --- | --- | --- | --- | --- | --- | --- | --- | --- | --- | --- | --- | --- | --- | --- | --- | --- | --- | --- | --- | --- | --- |
| PG3 | m + 0 | 0.970 | 0.935 | 0.892 | 0.832 | 0.770 | 0.731 | 0.682 | 0.650 | 0.633 | 0.590 | 0.559 | 0.521 | 0.485 | 0.456 | 0.423 | 0.421 | 0.430 | 0.435 | 0.458 | 0.433 |
|  | m + 1 | 0.030 | 0.067 | 0.096 | 0.151 | 0.197 | 0.218 | 0.248 | 0.266 | 0.264 | 0.280 | 0.285 | 0.295 | 0.282 | 0.268 | 0.252 | 0.251 | 0.253 | 0.251 | 0.234 | 0.244 |
|  | m + 2 | −0.001 | −0.002 | 0.009 | 0.007 | 0.014 | 0.022 | 0.033 | 0.038 | 0.045 | 0.060 | 0.072 | 0.088 | 0.116 | 0.146 | 0.165 | 0.158 | 0.154 | 0.155 | 0.151 | 0.158 |
|  | m + 3 | 0.002 | 0.000 | 0.003 | 0.010 | 0.019 | 0.029 | 0.037 | 0.046 | 0.058 | 0.070 | 0.084 | 0.096 | 0.117 | 0.130 | 0.160 | 0.169 | 0.163 | 0.159 | 0.157 | 0.165 |
| DHAP | m + 0 | 1.005 | 0.956 | 0.907 | 0.854 | 0.779 | 0.753 | 0.734 | 0.674 | 0.654 | 0.621 | 0.598 | 0.561 | 0.512 | 0.502 | 0.435 | 0.468 | 0.420 | 0.428 | 0.422 | 0.404 |
|  | m + 1 | 0.004 | 0.055 | 0.097 | 0.139 | 0.196 | 0.201 | 0.208 | 0.243 | 0.246 | 0.256 | 0.253 | 0.261 | 0.273 | 0.248 | 0.259 | 0.244 | 0.252 | 0.256 | 0.254 | 0.259 |
|  | m + 2 | −0.011 | −0.009 | −0.008 | 0.001 | 0.011 | 0.018 | 0.027 | 0.040 | 0.048 | 0.060 | 0.072 | 0.087 | 0.110 | 0.128 | 0.164 | 0.150 | 0.173 | 0.167 | 0.173 | 0.176 |
|  | m + 3 | 0.002 | −0.002 | 0.004 | 0.006 | 0.014 | 0.028 | 0.031 | 0.043 | 0.051 | 0.064 | 0.077 | 0.091 | 0.105 | 0.122 | 0.142 | 0.138 | 0.156 | 0.149 | 0.152 | 0.161 |
| Rul5P C3−C5 | m + 0 | 0.983 | 0.978 | 0.962 | 0.948 | 0.921 | 0.905 | 0.885 | 0.871 | 0.850 | 0.828 | 0.793 | 0.769 | 0.717 | 0.677 | 0.643 | 0.624 | 0.617 | 0.596 | 0.592 | 0.591 |
|  | m + 1 | 0.014 | 0.017 | 0.030 | 0.034 | 0.047 | 0.045 | 0.058 | 0.070 | 0.076 | 0.091 | 0.114 | 0.127 | 0.150 | 0.175 | 0.184 | 0.193 | 0.195 | 0.210 | 0.211 | 0.217 |
|  | m + 2 | 0.003 | 0.005 | 0.009 | 0.018 | 0.032 | 0.049 | 0.058 | 0.059 | 0.074 | 0.081 | 0.093 | 0.104 | 0.132 | 0.158 | 0.173 | 0.184 | 0.187 | 0.194 | 0.197 | 0.192 |
| Fru6P C4−C6 | m + 0 | 0.936 | 0.918 | 0.890 | 0.855 | 0.823 | 0.790 | 0.765 | 0.746 | 0.725 | 0.688 | 0.666 | 0.635 | 0.595 | 0.560 | 0.527 | 0.535 | 0.523 | 0.515 | 0.505 | 0.501 |
|  | m + 1 | 0.032 | 0.044 | 0.065 | 0.086 | 0.109 | 0.120 | 0.134 | 0.144 | 0.152 | 0.164 | 0.167 | 0.178 | 0.183 | 0.179 | 0.170 | 0.159 | 0.165 | 0.165 | 0.167 | 0.168 |
|  | m + 2 | 0.029 | 0.030 | 0.032 | 0.036 | 0.035 | 0.045 | 0.048 | 0.048 | 0.054 | 0.066 | 0.074 | 0.082 | 0.101 | 0.121 | 0.141 | 0.138 | 0.140 | 0.142 | 0.148 | 0.150 |
|  | m + 3 | 0.002 | 0.008 | 0.014 | 0.023 | 0.034 | 0.045 | 0.053 | 0.062 | 0.069 | 0.082 | 0.093 | 0.105 | 0.120 | 0.140 | 0.162 | 0.169 | 0.173 | 0.178 | 0.180 | 0.181 |
| Glc6P C3−C6 | m + 0 | 0.932 | 0.921 | 0.905 | 0.880 | 0.856 | 0.836 | 0.816 | 0.796 | 0.780 | 0.749 | 0.722 | 0.691 | 0.653 | 0.617 | 0.587 | 0.581 | 0.571 | 0.564 | 0.552 | 0.548 |
|  | m + 1 | 0.033 | 0.037 | 0.039 | 0.048 | 0.058 | 0.065 | 0.074 | 0.083 | 0.090 | 0.102 | 0.111 | 0.121 | 0.131 | 0.134 | 0.129 | 0.127 | 0.129 | 0.130 | 0.135 | 0.136 |
|  | m + 2 | 0.022 | 0.020 | 0.021 | 0.022 | 0.022 | 0.024 | 0.025 | 0.027 | 0.030 | 0.036 | 0.042 | 0.050 | 0.060 | 0.074 | 0.083 | 0.084 | 0.087 | 0.089 | 0.092 | 0.094 |
|  | m + 3 | 0.001 | 0.002 | 0.002 | 0.004 | 0.005 | 0.007 | 0.009 | 0.012 | 0.014 | 0.019 | 0.025 | 0.032 | 0.042 | 0.055 | 0.069 | 0.071 | 0.075 | 0.078 | 0.081 | 0.083 |
|  | m + 4 | 0.011 | 0.021 | 0.033 | 0.046 | 0.059 | 0.068 | 0.076 | 0.082 | 0.086 | 0.094 | 0.100 | 0.105 | 0.113 | 0.120 | 0.132 | 0.137 | 0.138 | 0.139 | 0.139 | 0.139 |
| Sed7P C4−C7 | m + 0 | 0.944 | 0.939 | 0.933 | 0.921 | 0.901 | 0.875 | 0.848 | 0.822 | 0.798 | 0.744 | 0.695 | 0.644 | 0.574 | 0.502 | 0.443 | 0.447 | 0.435 | 0.432 | 0.422 | 0.416 |
|  | m + 1 | 0.035 | 0.039 | 0.044 | 0.055 | 0.066 | 0.086 | 0.102 | 0.115 | 0.126 | 0.153 | 0.172 | 0.190 | 0.207 | 0.218 | 0.209 | 0.203 | 0.205 | 0.203 | 0.206 | 0.209 |
|  | m + 2 | 0.017 | 0.018 | 0.019 | 0.019 | 0.023 | 0.023 | 0.027 | 0.032 | 0.037 | 0.047 | 0.059 | 0.071 | 0.092 | 0.115 | 0.137 | 0.131 | 0.135 | 0.137 | 0.140 | 0.142 |
|  | m + 3 | 0.001 | 0.001 | 0.000 | 0.002 | 0.003 | 0.006 | 0.009 | 0.012 | 0.017 | 0.026 | 0.036 | 0.048 | 0.068 | 0.093 | 0.120 | 0.121 | 0.126 | 0.128 | 0.131 | 0.132 |
|  | m + 4 | 0.002 | 0.003 | 0.004 | 0.004 | 0.008 | 0.010 | 0.015 | 0.018 | 0.022 | 0.031 | 0.038 | 0.046 | 0.059 | 0.072 | 0.091 | 0.098 | 0.098 | 0.100 | 0.101 | 0.101 |
| Treh | m + 0 | 0.942 | 0.942 | 0.942 | 0.942 | 0.943 | 0.941 | 0.942 | 0.942 | 0.942 | 0.941 | 0.939 | 0.940 | 0.934 | 0.935 | 0.910 | 0.840 | 0.811 | 0.675 | 0.462 | 0.318 |
|  | m + 1 | 0.057 | 0.057 | 0.057 | 0.057 | 0.057 | 0.058 | 0.057 | 0.057 | 0.057 | 0.057 | 0.058 | 0.058 | 0.059 | 0.061 | 0.067 | 0.088 | 0.133 | 0.210 | 0.330 | 0.410 |
|  | m + 2 | 0.000 | −0.001 | 0.000 | 0.000 | −0.001 | 0.000 | 0.000 | 0.000 | 0.000 | 0.000 | 0.000 | 0.000 | 0.001 | 0.001 | 0.002 | 0.007 | 0.017 | 0.035 | 0.065 | 0.085 |

**Table S5A.** *Cont.*

|  | **Time (h)** | **0.001** | **0.003** | **0.006** | **0.008** | **0.011** | **0.014** | **0.017** | **0.019** | **0.022** | **0.028** | **0.033** | **0.042** | **0.056** | **0.083** | **0.167** | **0.500** | **1.000** | **2.000** | **4.000** | **6.000** |
| --- | --- | --- | --- | --- | --- | --- | --- | --- | --- | --- | --- | --- | --- | --- | --- | --- | --- | --- | --- | --- | --- |
| Treh | m + 3 | 0.000 | 0.000 | 0.000 | 0.000 | 0.000 | 0.000 | 0.000 | 0.000 | 0.000 | 0.000 | 0.000 | 0.000 | 0.000 | 0.000 | 0.001 | 0.005 | 0.011 | 0.022 | 0.040 | 0.053 |
|  | m + 4 | 0.001 | 0.001 | 0.001 | 0.001 | 0.001 | 0.001 | 0.000 | 0.001 | 0.000 | 0.001 | 0.001 | 0.000 | 0.001 | 0.001 | 0.001 | 0.004 | 0.008 | 0.017 | 0.030 | 0.040 |
|  | m + 5 | 0.000 | 0.000 | 0.000 | 0.000 | 0.000 | 0.000 | 0.000 | 0.000 | 0.000 | 0.000 | 0.000 | 0.000 | 0.000 | 0.000 | 0.001 | 0.003 | 0.003 | 0.007 | 0.013 | 0.018 |
|  | m + 6 | 0.000 | 0.001 | 0.000 | 0.000 | 0.000 | 0.001 | 0.000 | 0.000 | 0.000 | 0.001 | 0.003 | 0.000 | 0.005 | 0.001 | 0.016 | 0.052 | 0.016 | 0.034 | 0.060 | 0.076 |
| FBP C4−C6 | m + 0 | 0.940 | 0.909 | 0.876 | 0.819 | 0.774 | 0.735 | 0.697 | 0.658 | 0.650 | 0.596 | 0.578 | 0.550 | 0.511 | 0.500 | 0.452 | 0.459 | 0.447 | 0.434 | 0.442 | 0.446 |
|  | m + 1 | 0.042 | 0.059 | 0.107 | 0.139 | 0.167 | 0.191 | 0.207 | 0.218 | 0.214 | 0.241 | 0.242 | 0.243 | 0.244 | 0.221 | 0.219 | 0.203 | 0.210 | 0.209 | 0.216 | 0.197 |
|  | m + 2 | 0.019 | 0.027 | 0.012 | 0.033 | 0.053 | 0.044 | 0.050 | 0.068 | 0.077 | 0.095 | 0.092 | 0.112 | 0.135 | 0.159 | 0.170 | 0.167 | 0.181 | 0.178 | 0.181 | 0.192 |
|  | m + 3 | −0.001 | 0.006 | 0.005 | 0.008 | 0.006 | 0.031 | 0.046 | 0.056 | 0.060 | 0.068 | 0.087 | 0.095 | 0.111 | 0.120 | 0.159 | 0.171 | 0.161 | 0.179 | 0.161 | 0.165 |
| Pyr | m + 0 | 0.983 | 0.982 | 0.972 | 0.966 | 0.958 | 0.946 | 0.931 | 0.906 | 0.904 | 0.895 | 0.883 | 0.886 | 0.878 | 0.859 | 0.823 | 0.800 | 0.801 | 0.801 | 0.785 | 0.781 |
|  | m + 1 | 0.006 | 0.024 | 0.032 | 0.040 | 0.045 | 0.059 | 0.065 | 0.078 | 0.077 | 0.079 | 0.082 | 0.080 | 0.079 | 0.086 | 0.091 | 0.088 | 0.084 | 0.099 | 0.102 | 0.103 |
|  | m + 2 | 0.000 | −0.007 | −0.004 | −0.005 | −0.002 | −0.008 | −0.003 | 0.008 | 0.010 | 0.014 | 0.021 | 0.017 | 0.022 | 0.029 | 0.041 | 0.042 | 0.042 | 0.053 | 0.057 | 0.053 |
|  | m + 3 | 0.011 | 0.002 | −0.001 | −0.001 | −0.001 | 0.004 | 0.006 | 0.008 | 0.009 | 0.012 | 0.015 | 0.017 | 0.021 | 0.026 | 0.037 | 0.042 | 0.037 | 0.047 | 0.056 | 0.073 |
| Ala | m + 0 | 0.976 | 0.973 | 0.969 | 0.962 | 0.954 | 0.942 | 0.927 | 0.912 | 0.901 | 0.868 | 0.835 | 0.796 | 0.727 | 0.644 | 0.493 | 0.470 | 0.452 | 0.456 | 0.450 | 0.444 |
|  | m + 1 | 0.026 | 0.029 | 0.032 | 0.039 | 0.046 | 0.055 | 0.067 | 0.077 | 0.085 | 0.106 | 0.126 | 0.147 | 0.179 | 0.209 | 0.240 | 0.237 | 0.245 | 0.244 | 0.244 | 0.254 |
|  | m + 2 | −0.002 | −0.002 | −0.002 | −0.001 | 0.000 | 0.001 | 0.002 | 0.004 | 0.006 | 0.012 | 0.018 | 0.028 | 0.046 | 0.073 | 0.136 | 0.145 | 0.156 | 0.155 | 0.160 | 0.169 |
|  | m + 3 | 0.000 | 0.000 | 0.000 | 0.000 | 0.001 | 0.002 | 0.004 | 0.006 | 0.008 | 0.014 | 0.021 | 0.030 | 0.049 | 0.074 | 0.131 | 0.147 | 0.147 | 0.145 | 0.146 | 0.153 |
| Glut | m + 0 | 0.958 | 0.953 | 0.953 | 0.953 | 0.953 | 0.951 | 0.951 | 0.947 | 0.948 | 0.942 | 0.937 | 0.930 | 0.912 | 0.886 | 0.793 | 0.566 | 0.377 | 0.238 | 0.174 | 0.156 |
|  | m + 1 | 0.044 | 0.049 | 0.047 | 0.049 | 0.048 | 0.049 | 0.051 | 0.053 | 0.053 | 0.056 | 0.061 | 0.064 | 0.075 | 0.090 | 0.131 | 0.201 | 0.249 | 0.269 | 0.267 | 0.267 |
|  | m + 2 | −0.004 | −0.002 | 0.000 | −0.002 | −0.001 | −0.001 | −0.002 | −0.001 | −0.001 | 0.002 | 0.001 | 0.005 | 0.011 | 0.021 | 0.056 | 0.139 | 0.210 | 0.257 | 0.278 | 0.284 |
|  | m + 3 | 0.001 | 0.000 | −0.001 | −0.001 | −0.001 | −0.001 | 0.000 | 0.000 | 0.000 | −0.001 | 0.000 | 0.000 | 0.000 | 0.002 | 0.013 | 0.061 | 0.113 | 0.159 | 0.186 | 0.195 |
|  | m + 4 | 0.000 | 0.000 | 0.000 | 0.000 | 0.000 | 0.000 | 0.000 | 0.000 | 0.000 | 0.000 | 0.000 | 0.000 | 0.001 | 0.000 | 0.004 | 0.022 | 0.042 | 0.061 | 0.077 | 0.078 |
|  | m + 5 | 0.001 | 0.001 | 0.001 | 0.001 | 0.000 | 0.001 | 0.001 | 0.001 | 0.001 | 0.001 | 0.001 | 0.001 | 0.001 | 0.001 | 0.004 | 0.011 | 0.010 | 0.016 | 0.018 | 0.020 |
| Asp | m + 0 | 0.968 | 0.964 | 0.964 | 0.963 | 0.961 | 0.957 | 0.954 | 0.949 | 0.946 | 0.936 | 0.926 | 0.910 | 0.878 | 0.821 | 0.662 | 0.440 | 0.326 | 0.256 | 0.221 | 0.209 |
|  | m + 1 | 0.034 | 0.037 | 0.037 | 0.039 | 0.041 | 0.044 | 0.046 | 0.049 | 0.051 | 0.058 | 0.065 | 0.074 | 0.091 | 0.119 | 0.182 | 0.258 | 0.293 | 0.311 | 0.315 | 0.315 |
|  | m + 2 | −0.002 | −0.001 | −0.001 | −0.001 | −0.001 | −0.001 | 0.000 | 0.000 | 0.001 | 0.002 | 0.004 | 0.008 | 0.017 | 0.032 | 0.086 | 0.166 | 0.210 | 0.238 | 0.253 | 0.258 |
|  | m + 3 | −0.001 | 0.000 | 0.000 | 0.000 | 0.000 | 0.000 | 0.001 | 0.001 | 0.002 | 0.003 | 0.004 | 0.007 | 0.013 | 0.023 | 0.056 | 0.100 | 0.125 | 0.143 | 0.154 | 0.160 |
|  | m + 4 | 0.000 | 0.000 | 0.000 | 0.000 | 0.000 | 0.000 | 0.000 | 0.000 | 0.000 | 0.000 | 0.000 | 0.001 | 0.002 | 0.004 | 0.015 | 0.036 | 0.046 | 0.052 | 0.057 | 0.059 |

**Table S5B.** Measured mass isotopomers of *P. pastoris* X-33 Expressing Strain during the wash-in experiment.

|  | **Time (h)** | **0.001** | **0.003** | **0.006** | **0.008** | **0.011** | **0.014** | **0.017** | **0.019** | **0.022** | **0.028** | **0.033** | **0.042** | **0.056** | **0.083** | **0.167** | **0.500** | **1.000** | **2.000** | **3.000** | **4.000** |
| --- | --- | --- | --- | --- | --- | --- | --- | --- | --- | --- | --- | --- | --- | --- | --- | --- | --- | --- | --- | --- | --- |
| FUM | m + 0 | 0.963 | 0.961 | 0.963 | 0.957 | 0.962 | 0.958 | 0.953 | 0.950 | 0.951 | 0.946 | 0.936 | 0.923 | 0.911 | 0.876 | 0.769 | 0.554 | 0.419 | 0.316 | 0.280 | 0.277 |
|  | m + 1 | 0.033 | 0.037 | 0.037 | 0.040 | 0.035 | 0.038 | 0.044 | 0.045 | 0.044 | 0.048 | 0.055 | 0.059 | 0.065 | 0.086 | 0.131 | 0.212 | 0.261 | 0.287 | 0.290 | 0.290 |
|  | m + 2 | 0.002 | 0.000 | −0.001 | 0.001 | 0.002 | 0.003 | 0.002 | 0.005 | 0.005 | 0.005 | 0.006 | 0.014 | 0.015 | 0.024 | 0.059 | 0.136 | 0.187 | 0.230 | 0.243 | 0.242 |
|  | m + 3 | 0.001 | 0.001 | 0.000 | 0.001 | 0.001 | 0.001 | 0.001 | 0.001 | 0.000 | 0.002 | 0.003 | 0.004 | 0.007 | 0.013 | 0.034 | 0.077 | 0.103 | 0.124 | 0.136 | 0.138 |
|  | m + 4 | 0.001 | 0.000 | 0.000 | 0.000 | 0.000 | 0.000 | 0.000 | 0.000 | 0.000 | 0.000 | 0.000 | −0.001 | 0.001 | 0.001 | 0.007 | 0.021 | 0.030 | 0.042 | 0.050 | 0.053 |
| SUCC | m + 0 | 0.964 | 0.964 | 0.964 | 0.964 | 0.963 | 0.964 | 0.963 | 0.963 | 0.962 | 0.960 | 0.956 | 0.953 | 0.944 | 0.924 | 0.863 | 0.686 | 0.571 | 0.408 | 0.377 | 0.364 |
|  | m + 1 | 0.039 | 0.038 | 0.039 | 0.040 | 0.041 | 0.040 | 0.040 | 0.040 | 0.041 | 0.043 | 0.045 | 0.047 | 0.053 | 0.066 | 0.095 | 0.178 | 0.219 | 0.279 | 0.283 | 0.284 |
|  | m + 2 | −0.003 | −0.002 | −0.003 | −0.004 | −0.004 | −0.003 | −0.003 | −0.004 | −0.003 | −0.003 | −0.001 | 0.001 | 0.003 | 0.009 | 0.036 | 0.098 | 0.143 | 0.203 | 0.216 | 0.223 |
|  | m + 3 | −0.001 | −0.001 | −0.001 | −0.001 | −0.001 | −0.001 | −0.001 | 0.000 | 0.000 | −0.001 | −0.001 | −0.001 | −0.001 | 0.000 | 0.006 | 0.032 | 0.054 | 0.087 | 0.098 | 0.102 |
|  | m + 4 | 0.001 | 0.001 | 0.000 | 0.001 | 0.000 | 0.000 | 0.001 | 0.000 | 0.000 | 0.000 | 0.001 | 0.000 | 0.000 | 0.000 | 0.001 | 0.006 | 0.013 | 0.023 | 0.026 | 0.027 |
| MAL | m + 0 | 0.957 | 0.957 | 0.955 | 0.955 | 0.955 | 0.950 | 0.951 | 0.947 | 0.942 | 0.939 | 0.934 | 0.922 | 0.905 | 0.862 | 0.750 | 0.559 | 0.382 | 0.314 | 0.276 | 0.271 |
|  | m + 1 | 0.041 | 0.042 | 0.045 | 0.044 | 0.044 | 0.047 | 0.048 | 0.050 | 0.056 | 0.057 | 0.059 | 0.066 | 0.077 | 0.098 | 0.147 | 0.229 | 0.280 | 0.305 | 0.312 | 0.310 |
|  | m + 2 | −0.001 | −0.002 | −0.003 | −0.001 | −0.001 | 0.000 | −0.002 | −0.001 | −0.001 | 0.000 | 0.002 | 0.005 | 0.008 | 0.021 | 0.054 | 0.133 | 0.185 | 0.224 | 0.239 | 0.241 |
|  | m + 3 | 0.002 | 0.002 | 0.002 | 0.001 | 0.002 | 0.001 | 0.002 | 0.002 | 0.001 | 0.003 | 0.004 | 0.004 | 0.007 | 0.014 | 0.038 | 0.052 | 0.111 | 0.106 | 0.118 | 0.120 |
|  | m + 4 | 0.001 | 0.001 | 0.001 | 0.001 | 0.001 | 0.001 | 0.001 | 0.001 | 0.002 | 0.001 | 0.001 | 0.002 | 0.003 | 0.004 | 0.011 | 0.028 | 0.041 | 0.051 | 0.055 | 0.058 |
| αKG | m + 0 | 0.969 | 0.963 | 0.963 | 0.961 | 0.959 | 0.963 | 0.959 | 0.958 | 0.966 | 0.956 | 0.958 | 0.969 | 0.947 | 0.929 | 0.873 | 0.665 | 0.486 | 0.320 | 0.292 | 0.281 |
|  | m + 1 | 0.029 | 0.033 | 0.027 | 0.033 | 0.033 | 0.031 | 0.035 | 0.036 | 0.030 | 0.035 | 0.035 | 0.027 | 0.045 | 0.055 | 0.075 | 0.149 | 0.200 | 0.246 | 0.241 | 0.246 |
|  | m + 2 | −0.007 | −0.004 | −0.004 | −0.005 | −0.002 | −0.006 | −0.003 | −0.003 | −0.009 | −0.001 | −0.001 | −0.008 | 0.001 | 0.010 | 0.037 | 0.115 | 0.176 | 0.232 | 0.245 | 0.240 |
|  | m + 3 | 0.001 | 0.003 | 0.004 | 0.003 | 0.004 | 0.003 | 0.003 | 0.004 | 0.001 | 0.005 | 0.004 | 0.005 | 0.003 | 0.003 | 0.009 | 0.052 | 0.097 | 0.137 | 0.151 | 0.156 |
|  | m + 4 | 0.008 | 0.006 | 0.009 | 0.007 | 0.006 | 0.007 | 0.005 | 0.005 | 0.010 | 0.004 | 0.004 | 0.006 | 0.003 | 0.003 | 0.004 | 0.016 | 0.035 | 0.053 | 0.059 | 0.064 |
|  | m + 5 | 0.001 | 0.000 | 0.001 | 0.001 | 0.001 | 0.001 | 0.000 | 0.001 | 0.002 | 0.001 | 0.000 | 0.000 | 0.001 | 0.001 | 0.002 | 0.004 | 0.007 | 0.012 | 0.013 | 0.014 |
| PG2 | m + 0 | 0.970 | 0.929 | 0.866 | 0.811 | 0.780 | 0.743 | 0.736 | 0.697 | 0.672 | 0.660 | 0.629 | 0.597 | 0.551 | 0.510 | 0.472 | 0.475 | 0.459 | 0.461 | 0.461 | 0.459 |
|  | m + 1 | 0.027 | 0.067 | 0.119 | 0.177 | 0.192 | 0.219 | 0.226 | 0.238 | 0.242 | 0.230 | 0.248 | 0.236 | 0.246 | 0.249 | 0.241 | 0.235 | 0.248 | 0.251 | 0.234 | 0.233 |
|  | m + 2 | 0.005 | 0.000 | 0.008 | 0.001 | 0.006 | 0.011 | 0.014 | 0.029 | 0.036 | 0.049 | 0.053 | 0.077 | 0.105 | 0.114 | 0.139 | 0.138 | 0.142 | 0.132 | 0.152 | 0.146 |
|  | m + 3 | −0.002 | 0.003 | 0.007 | 0.011 | 0.022 | 0.028 | 0.024 | 0.036 | 0.050 | 0.061 | 0.070 | 0.090 | 0.098 | 0.127 | 0.147 | 0.153 | 0.151 | 0.156 | 0.153 | 0.163 |
| PG3 | m + 0 | 0.964 | 0.931 | 0.872 | 0.818 | 0.775 | 0.747 | 0.721 | 0.701 | 0.689 | 0.651 | 0.629 | 0.601 | 0.563 | 0.513 | 0.476 | 0.474 | 0.463 | 0.466 | 0.450 | 0.461 |
|  | m + 1 | 0.036 | 0.072 | 0.124 | 0.167 | 0.198 | 0.214 | 0.222 | 0.231 | 0.238 | 0.244 | 0.249 | 0.258 | 0.251 | 0.251 | 0.244 | 0.223 | 0.231 | 0.229 | 0.234 | 0.231 |

**Table S5B.** *Cont.*

|  | **Time (h)** | **0.001** | **0.003** | **0.006** | **0.008** | **0.011** | **0.014** | **0.017** | **0.019** | **0.022** | **0.028** | **0.033** | **0.042** | **0.056** | **0.083** | **0.167** | **0.500** | **1.000** | **2.000** | **3.000** | **4.000** |
| --- | --- | --- | --- | --- | --- | --- | --- | --- | --- | --- | --- | --- | --- | --- | --- | --- | --- | --- | --- | --- | --- |
| PG3 | m + 2 | −0.001 | −0.002 | 0.002 | 0.007 | 0.013 | 0.016 | 0.026 | 0.030 | 0.035 | 0.044 | 0.054 | 0.072 | 0.086 | 0.116 | 0.144 | 0.151 | 0.151 | 0.148 | 0.154 | 0.148 |
|  | m + 3 | 0.001 | 0.000 | 0.002 | 0.007 | 0.015 | 0.023 | 0.032 | 0.037 | 0.039 | 0.061 | 0.068 | 0.069 | 0.099 | 0.120 | 0.135 | 0.152 | 0.155 | 0.157 | 0.161 | 0.160 |
| DHAP | m + 0 | 0.988 | 0.923 | 0.827 | 0.789 | 0.738 | 0.714 | 0.696 | 0.650 | 0.611 | 0.619 | 0.578 | 0.533 | 0.513 | 0.438 | 0.404 | 0.371 | 0.389 | 0.388 | 0.392 | 0.398 |
|  | m + 1 | 0.036 | 0.092 | 0.178 | 0.210 | 0.244 | 0.256 | 0.259 | 0.293 | 0.315 | 0.281 | 0.301 | 0.315 | 0.286 | 0.301 | 0.284 | 0.289 | 0.288 | 0.273 | 0.281 | 0.283 |
|  | m + 2 | −0.023 | −0.011 | −0.007 | −0.002 | 0.006 | 0.011 | 0.022 | 0.031 | 0.039 | 0.050 | 0.059 | 0.087 | 0.111 | 0.149 | 0.175 | 0.180 | 0.180 | 0.183 | 0.182 | 0.181 |
|  | m + 3 | −0.002 | −0.004 | 0.002 | 0.003 | 0.012 | 0.019 | 0.023 | 0.026 | 0.036 | 0.050 | 0.061 | 0.065 | 0.090 | 0.112 | 0.137 | 0.160 | 0.143 | 0.157 | 0.144 | 0.138 |
| Ribu5P | m + 0 | 0.981 | 0.972 | 0.961 | 0.940 | 0.925 | 0.911 | 0.893 | 0.876 | 0.859 | 0.834 | 0.808 | 0.776 | 0.738 | 0.682 | 0.622 | 0.604 | 0.605 | 0.596 | 0.591 | 0.592 |
|  | m + 1 | 0.018 | 0.024 | 0.033 | 0.045 | 0.050 | 0.057 | 0.066 | 0.075 | 0.083 | 0.096 | 0.108 | 0.123 | 0.143 | 0.174 | 0.221 | 0.225 | 0.222 | 0.227 | 0.222 | 0.231 |
|  | m + 2 | 0.001 | 0.004 | 0.007 | 0.016 | 0.024 | 0.032 | 0.041 | 0.049 | 0.059 | 0.070 | 0.084 | 0.100 | 0.119 | 0.143 | 0.157 | 0.171 | 0.173 | 0.176 | 0.177 | 0.177 |
| Fru6P C4−C6 | m + 0 | 0.937 | 0.919 | 0.888 | 0.856 | 0.825 | 0.803 | 0.783 | 0.761 | 0.744 | 0.716 | 0.691 | 0.657 | 0.616 | 0.567 | 0.521 | 0.514 | 0.509 | 0.503 | 0.499 | 0.501 |
|  | m + 1 | 0.038 | 0.051 | 0.076 | 0.097 | 0.116 | 0.128 | 0.135 | 0.145 | 0.151 | 0.160 | 0.169 | 0.179 | 0.187 | 0.189 | 0.193 | 0.185 | 0.187 | 0.188 | 0.187 | 0.187 |
|  | m + 2 | 0.024 | 0.027 | 0.027 | 0.029 | 0.032 | 0.034 | 0.037 | 0.042 | 0.047 | 0.053 | 0.059 | 0.071 | 0.087 | 0.112 | 0.126 | 0.129 | 0.130 | 0.131 | 0.135 | 0.132 |
|  | m + 3 | 0.001 | 0.003 | 0.009 | 0.018 | 0.027 | 0.035 | 0.045 | 0.051 | 0.058 | 0.071 | 0.081 | 0.093 | 0.109 | 0.132 | 0.160 | 0.172 | 0.175 | 0.178 | 0.179 | 0.180 |
| Glc6P C3−C6 | m + 0 | 0.932 | 0.925 | 0.909 | 0.888 | 0.868 | 0.842 | 0.823 | 0.803 | 0.783 | 0.752 | 0.724 | 0.689 | 0.645 | 0.594 | 0.544 | 0.532 | 0.522 | 0.519 | 0.517 | 0.517 |
|  | m + 1 | 0.036 | 0.037 | 0.044 | 0.054 | 0.062 | 0.076 | 0.085 | 0.094 | 0.105 | 0.117 | 0.128 | 0.140 | 0.150 | 0.156 | 0.151 | 0.147 | 0.152 | 0.150 | 0.152 | 0.150 |
|  | m + 2 | 0.022 | 0.023 | 0.022 | 0.023 | 0.022 | 0.025 | 0.025 | 0.029 | 0.030 | 0.035 | 0.041 | 0.051 | 0.064 | 0.082 | 0.100 | 0.101 | 0.102 | 0.104 | 0.104 | 0.105 |
|  | m + 3 | 0.001 | 0.001 | 0.001 | 0.002 | 0.005 | 0.006 | 0.008 | 0.011 | 0.013 | 0.019 | 0.024 | 0.032 | 0.043 | 0.061 | 0.083 | 0.092 | 0.096 | 0.097 | 0.098 | 0.097 |
|  | m + 4 | 0.010 | 0.014 | 0.023 | 0.033 | 0.043 | 0.051 | 0.058 | 0.063 | 0.069 | 0.077 | 0.083 | 0.089 | 0.097 | 0.107 | 0.122 | 0.128 | 0.128 | 0.129 | 0.130 | 0.131 |
| Sed7P | m + 0 | 0.944 | 0.941 | 0.936 | 0.925 | 0.912 | 0.883 | 0.869 | 0.849 | 0.827 | 0.788 | 0.751 | 0.696 | 0.637 | 0.543 | 0.451 | 0.434 | 0.432 | 0.423 | 0.418 | 0.421 |
|  | m + 1 | 0.029 | 0.039 | 0.041 | 0.054 | 0.064 | 0.080 | 0.093 | 0.103 | 0.111 | 0.135 | 0.151 | 0.173 | 0.190 | 0.209 | 0.207 | 0.199 | 0.203 | 0.208 | 0.203 | 0.202 |
|  | m + 2 | 0.020 | 0.015 | 0.016 | 0.015 | 0.016 | 0.021 | 0.022 | 0.025 | 0.029 | 0.037 | 0.043 | 0.054 | 0.073 | 0.102 | 0.133 | 0.141 | 0.135 | 0.137 | 0.142 | 0.140 |
|  | m + 3 | 0.001 | 0.000 | 0.001 | 0.001 | 0.003 | 0.004 | 0.006 | 0.008 | 0.011 | 0.016 | 0.025 | 0.035 | 0.052 | 0.080 | 0.118 | 0.129 | 0.131 | 0.135 | 0.137 | 0.136 |
|  | m + 4 | 0.005 | 0.004 | 0.005 | 0.004 | 0.006 | 0.012 | 0.010 | 0.015 | 0.022 | 0.023 | 0.031 | 0.042 | 0.048 | 0.065 | 0.090 | 0.097 | 0.098 | 0.097 | 0.101 | 0.101 |
| Treh | m + 0 | 0.941 | 0.941 | 0.941 | 0.941 | 0.941 | 0.941 | 0.941 | 0.941 | 0.940 | 0.941 | 0.940 | 0.939 | 0.936 | 0.929 | 0.907 | 0.843 | 0.769 | 0.646 | 0.547 | 0.476 |
|  | m + 1 | 0.058 | 0.058 | 0.058 | 0.058 | 0.058 | 0.058 | 0.058 | 0.058 | 0.058 | 0.058 | 0.059 | 0.059 | 0.061 | 0.066 | 0.078 | 0.112 | 0.151 | 0.217 | 0.270 | 0.308 |
|  | m + 2 | 0.000 | 0.000 | 0.000 | 0.000 | 0.000 | 0.000 | 0.000 | 0.000 | 0.000 | 0.000 | 0.000 | 0.000 | 0.000 | 0.001 | 0.005 | 0.014 | 0.025 | 0.044 | 0.060 | 0.071 |
|  | m + 3 | 0.000 | 0.000 | 0.000 | 0.000 | 0.000 | 0.000 | 0.000 | 0.000 | 0.000 | 0.000 | 0.000 | 0.000 | 0.000 | 0.001 | 0.003 | 0.009 | 0.017 | 0.029 | 0.039 | 0.046 |

**Table S5B.** *Cont.*

|  | **Time (h)** | **0.001** | **0.003** | **0.006** | **0.008** | **0.011** | **0.014** | **0.017** | **0.019** | **0.022** | **0.028** | **0.033** | **0.042** | **0.056** | **0.083** | **0.167** | **0.500** | **1.000** | **2.000** | **3.000** | **4.000** |
| --- | --- | --- | --- | --- | --- | --- | --- | --- | --- | --- | --- | --- | --- | --- | --- | --- | --- | --- | --- | --- | --- |
| Treh | m + 4 | 0.001 | 0.001 | 0.001 | 0.001 | 0.001 | 0.001 | 0.001 | 0.001 | 0.001 | 0.001 | 0.001 | 0.001 | 0.001 | 0.001 | 0.002 | 0.007 | 0.013 | 0.022 | 0.030 | 0.035 |
|  | m + 5 | 0.000 | 0.000 | 0.000 | 0.000 | 0.000 | 0.000 | 0.000 | 0.000 | 0.000 | 0.000 | 0.000 | 0.000 | 0.000 | 0.000 | 0.001 | 0.003 | 0.006 | 0.009 | 0.012 | 0.015 |
|  | m + 6 | 0.000 | 0.000 | 0.000 | 0.000 | 0.000 | 0.000 | 0.000 | 0.000 | 0.001 | 0.000 | 0.000 | 0.001 | 0.001 | 0.002 | 0.004 | 0.011 | 0.019 | 0.032 | 0.042 | 0.049 |
| FBP C4−C6 | m + 0 | 1.010 | 1.010 | 0.989 | 0.952 | 0.884 | 0.825 | 0.844 | 0.814 | 0.742 | 0.776 | 0.697 | 0.681 | 0.660 | 0.562 | 0.521 | 0.491 | 0.484 | 0.466 | 0.455 | 0.485 |
|  | m + 1 | 0.098 | 0.071 | 0.099 | 0.126 | 0.167 | 0.208 | 0.234 | 0.265 | 0.230 | 0.232 | 0.239 | 0.246 | 0.239 | 0.262 | 0.248 | 0.230 | 0.248 | 0.252 | 0.245 | 0.242 |
|  | m + 2 | −0.103 | −0.060 | −0.056 | −0.077 | −0.070 | −0.051 | −0.091 | −0.085 | −0.021 | −0.054 | 0.019 | 0.016 | 0.013 | 0.068 | 0.107 | 0.127 | 0.121 | 0.134 | 0.146 | 0.140 |
|  | m + 3 | −0.004 | −0.021 | −0.033 | 0.000 | 0.019 | 0.019 | 0.012 | 0.006 | 0.049 | 0.046 | 0.045 | 0.057 | 0.088 | 0.108 | 0.124 | 0.152 | 0.146 | 0.148 | 0.154 | 0.134 |
| Pyr | m + 0 | 0.944 | 0.958 | 0.953 | 0.939 | 0.938 | 0.927 | 0.931 | 0.922 | 0.924 | 0.915 | 0.904 | 0.902 | 0.876 | 0.854 | 0.820 | 0.794 | 0.792 | 0.778 | 0.788 | 0.784 |
|  | m + 1 | 0.030 | 0.033 | 0.039 | 0.049 | 0.052 | 0.062 | 0.059 | 0.061 | 0.064 | 0.063 | 0.071 | 0.068 | 0.080 | 0.079 | 0.099 | 0.106 | 0.103 | 0.109 | 0.104 | 0.108 |
|  | m + 2 | 0.009 | 0.006 | 0.006 | 0.010 | 0.007 | 0.008 | 0.004 | 0.010 | 0.005 | 0.012 | 0.015 | 0.017 | 0.024 | 0.041 | 0.042 | 0.052 | 0.063 | 0.066 | 0.067 | 0.067 |
|  | m + 3 | 0.017 | 0.003 | 0.002 | 0.002 | 0.004 | 0.004 | 0.005 | 0.007 | 0.008 | 0.010 | 0.011 | 0.014 | 0.020 | 0.026 | 0.039 | 0.038 | 0.042 | 0.046 | 0.042 | 0.042 |
| Ala | m + 0 | 0.975 | 0.973 | 0.972 | 0.961 | 0.949 | 0.940 | 0.924 | 0.910 | 0.900 | 0.869 | 0.844 | 0.810 | 0.740 | 0.646 | 0.525 | 0.493 | 0.474 | 0.442 | 0.433 | 0.424 |
|  | m + 1 | 0.027 | 0.029 | 0.031 | 0.040 | 0.051 | 0.059 | 0.071 | 0.082 | 0.087 | 0.110 | 0.123 | 0.142 | 0.177 | 0.212 | 0.235 | 0.232 | 0.240 | 0.251 | 0.255 | 0.258 |
|  | m + 2 | −0.002 | −0.002 | −0.002 | −0.001 | −0.001 | 0.000 | 0.001 | 0.003 | 0.004 | 0.009 | 0.014 | 0.021 | 0.038 | 0.070 | 0.125 | 0.143 | 0.149 | 0.161 | 0.163 | 0.166 |
|  | m + 3 | 0.000 | 0.000 | 0.000 | 0.000 | 0.001 | 0.001 | 0.004 | 0.005 | 0.008 | 0.013 | 0.018 | 0.027 | 0.044 | 0.072 | 0.114 | 0.132 | 0.137 | 0.146 | 0.149 | 0.152 |
| Glu | m + 0 | 0.957 | 0.957 | 0.962 | 0.958 | 0.956 | 0.959 | 0.957 | 0.953 | 0.952 | 0.948 | 0.946 | 0.938 | 0.924 | 0.901 | 0.801 | 0.562 | 0.380 | 0.240 | 0.194 | 0.187 |
|  | m + 1 | 0.046 | 0.046 | 0.042 | 0.047 | 0.048 | 0.049 | 0.045 | 0.049 | 0.052 | 0.053 | 0.054 | 0.060 | 0.069 | 0.080 | 0.130 | 0.207 | 0.249 | 0.266 | 0.269 | 0.262 |
|  | m + 2 | −0.002 | −0.003 | −0.005 | −0.005 | −0.004 | −0.009 | −0.002 | −0.002 | −0.003 | −0.001 | 0.001 | 0.003 | 0.006 | 0.017 | 0.053 | 0.143 | 0.208 | 0.255 | 0.271 | 0.273 |
|  | m + 3 | −0.001 | −0.001 | −0.001 | −0.001 | −0.001 | 0.000 | −0.001 | −0.001 | −0.001 | −0.001 | −0.001 | −0.002 | 0.000 | 0.002 | 0.012 | 0.061 | 0.112 | 0.161 | 0.178 | 0.184 |
|  | m + 4 | 0.000 | 0.000 | 0.001 | 0.000 | 0.000 | 0.000 | 0.000 | 0.001 | 0.000 | 0.000 | 0.001 | 0.001 | 0.001 | 0.000 | 0.004 | 0.022 | 0.043 | 0.063 | 0.071 | 0.077 |
|  | m + 5 | 0.000 | 0.000 | 0.000 | 0.000 | 0.000 | 0.001 | 0.000 | 0.000 | 0.000 | 0.000 | 0.000 | 0.000 | 0.000 | 0.000 | 0.001 | 0.005 | 0.010 | 0.015 | 0.017 | 0.017 |
| Asp | m + 0 | 0.966 | 0.966 | 0.967 | 0.965 | 0.961 | 0.963 | 0.954 | 0.951 | 0.946 | 0.939 | 0.929 | 0.913 | 0.884 | 0.826 | 0.674 | 0.481 | 0.371 | 0.294 | 0.268 | 0.264 |
|  | m + 1 | 0.036 | 0.036 | 0.035 | 0.037 | 0.040 | 0.040 | 0.046 | 0.049 | 0.052 | 0.056 | 0.064 | 0.073 | 0.091 | 0.120 | 0.180 | 0.255 | 0.290 | 0.308 | 0.311 | 0.310 |
|  | m + 2 | −0.002 | −0.002 | −0.002 | −0.002 | −0.002 | −0.004 | −0.001 | 0.000 | 0.000 | 0.003 | 0.003 | 0.007 | 0.014 | 0.030 | 0.081 | 0.167 | 0.209 | 0.239 | 0.249 | 0.250 |
|  | m + 3 | 0.000 | 0.000 | −0.001 | 0.000 | 0.000 | 0.001 | 0.000 | 0.001 | 0.001 | 0.003 | 0.004 | 0.006 | 0.011 | 0.021 | 0.021 | 0.062 | 0.086 | 0.106 | 0.116 | 0.117 |
|  | m + 4 | 0.000 | 0.000 | 0.000 | 0.000 | 0.000 | 0.000 | 0.000 | 0.000 | 0.000 | 0.000 | 0.000 | 0.000 | 0.001 | 0.003 | 0.013 | 0.035 | 0.044 | 0.053 | 0.057 | 0.059 |
