# Supplementary material for: Quantitative Metabolomics and Instationary 13C-Metabolic Flux Analysis Reveals Impact of Recombinant Protein Production on Trehalose and Energy Metabolism in Pichia pastoris
Source: Metabolites. 2014 May 5;4(2):281–99. doi: 10.3390/metabo4020281 (PMC4101507; doi:10.3390/metabo4020281)
Supplement: Supplementary File 1 — Supplementary Materials (ZIP, 2359 KB) [file metabolites-04-00281-s001.zip › metabolites-04-00281-supplementary/Supplementary file 6.docx]

Supplementary file 6

Metabolic models of the central carbon metabolism of *P. pastoris.* (S6.1) Stoichiometric model of the central carbon metabolism of *P. pastoris*, applied in the ^13^C-MFA. Note that CO_2_, O_2_ and cofactors were not used for flux balancing. (S6.2) Reaction network used for anNET analysis. (S6.3) Reaction-and atom transition network used in ^13^C flux analysis.

S6.1. Stochiometric Model applied in the ^13^C-MFA of P. pastoris

Methanol metabolism

1. Metoh → Form
2. Form → FOR + NADH
3. FOR + NAD^+^ → NADH + CO_2_
4. Xul5P + FOR + ATP → ADP + GA3P_per_ + DHA
5. GA3P_per_ → GA3P
6. DHA → GA3P

Glycolysis and gluconeogenesis pathways

1. Glc_ext_ + 2 ATP ↔ Glc6P + 2 ADP
2. Glc6P ↔ Fru6P
3. Fru6P + 1 ATP → FBP + 1ADP
4. FBP → Fru6P + Pi
5. FBP ↔ DHAP + GA3P
6. GA3P + ADP + Pi + NAD+ → PG3 + ATP + NADH
7. PG3 + ATP + NADH → GA3P + ADP + Pi + NAD^+^
8. PG3 ↔ Pep
9. Pep + ADP → Pyr + ATP
10. Pyr + NAD^+^ → ACCoA_mit_ + CO_2_ + NADH
11. Pyr + NAD^+^ → ACCoA_cyt_ + CO_2_ + NADH
12. Pyr + CO_2_ + ADP → OAA + ATP
13. 2 Glc6P + UTP + H_2_O → H^+^ +2 Pi + UDP + T6P
14. T6P + H_2_O → Pi + Treh
15. Treh + H_2_O → 2 Glc6P

Pentose phosphate pathway

1. Glc6P + 2 NADP^+^ → Rul5P + 2 NADPH + CO_2_
2. Rul5P ↔ Rib5P
3. Rul5P ↔ Xul5P
4. Rib5P + Xul5P ↔ Sed7P + GA3P
5. Sed7P + GA3P ↔ Fru6P + E4P
6. Xul5P + E4P ↔ Fru6P + GA3P

TCA cycle

1. ACCoA_mit_ + OAA → CIT
2. CIT → ICIT
3. ICIT + NAD^+^ → KG + CO_2_ + NADH
4. KG + NAD^+^ → SUCCoA + CO_2_ + NADH
5. SUCCoA + Pi + ADP → SUCC + ATP
6. SUCC + ATP → SUCCoA + ADP + Pi
7. SUCC + NAD^+^ → FUM + NADH
8. FUM + H_2_O ↔ MAL
9. MAL + NAD^+^ ↔ OAA + NADH
10. Asp + 2 ATP + H_2_O → FUM + 2 ADP + 2Pi

Biosynthesis of amino acids

Serine family

1. PG3 + Glu + NAD^+^ → Ser + KG + NADH +Pi
2. Ser + THF → Gly + MetTHF
3. Ser + ACCoA + H_2_S → Cys

Alanine family

1. Pyr + NADPH → Ala + NADP^+^
2. Pyr + Glu → KG
3. 2 Pyr + NADPH → Kval + CO_2_ + NADP^+^
4. Kval + Glu → Val + KG
5. Kval + ACCoA_mit_ + Glu + NAD^+^ → Leu + KG + NADH + CO_2_

Histidine family

1. Rib5P + ATP → PRPP + AMP
2. PRPP + ATP + Gln + 2 NAD^+^ → His + KG + Pi + 2 NADH

Aspartic family

1. OAA + Glu → Asp + KG
2. Asp + Gln + ATP → Asn + Glu + AMP
3. Asp + ATP + 2 NADPH → Ser + ADP + Pi + 2 NADP^+^
4. Thr + NADPH + Glu + Pyr → Ile + KG + NH_4_ + CO_2_ + NADP^+^
5. ACCoA + Ser + H_2_S + MTHF → Met + THF

Aromatic family

1. 2 Pep + E4P + ATP + NADPH → CHOR + ADP + 4 Pi
2. CHOR + Glu → Phe + KG + CO_2_
3. CHOR + Glu → Tyr + KG + NADH + CO_2_
4. CHOR + Gln + PRPP + Ser → Trp + Glu + Pyr + GA3P + CO_2_

Glutamic family

1. KG +NH_4_ + NADPH → Glu + NADP^+^ + H_2_O
2. Glut + ATP + NH_4_ → Gln + ADP + Pi
3. Glut + ATP + 2 NADPH → Pro + ADP + Pi
4. Gln + CO_2_ + 2 ATP → CaP + Glu + 2 ADP + Pi
5. Glu + ACCoA_mit_ + 4 ATP + NADPH + CaP + Asp → Arg + KG + 4 ADP + FUM + 5 Pi
6. 2 Glu + ACCoA_mit_+ 3 ATP + 2 NADPH + 2 NAD^+^ → Lys + KG + CO_2_ + 2 NADH + 2 NADP^+^

Biosynthesis and interconversion of one-carbon units

1. DHF + NADPH → THF + NADP^+^
2. Gly + THF + NAD^+^ → MTHF + NH^4+^ + NADH + CO_2_
3. MTHF + NADH → THF + NAD^+^

Transport reactions

1. ACCoA_cyt_ → ACCoA_mit_
2. NH^4+^_ext_ + ATP → NH^4+^_cyt_ + ADP + Pi
3. SO_4_^−2^_ext_ + ATP → SO_4_^−2^_cyt_ + ADP + Pi
4. Metal_ext_ + ATP → Metal_cyt_ + ADP + Pi

Respiratory chain

1. NADH + 0.5 O_2_ → NAD^+^ + H_2_O
2. ADP + Pi → ATP

Biomass synthesis

(1) **Protein synthesis** (Composition derived from the measured amino acid composition [1]. The energy needed to biosynthesize 1 C-mol of protein was derived from the synthesis of each amino acid and the protein polymerization value taken from [2].

X-33 Control Strain

1.(A) 0.148 Pyr + 0.083 NAD^+^ + 0.0423 NADPH + 1.21 ATP + 0.0072 Rib5P + 0.0147 E4P + 0.044 OAA+ 0.063 αKG_mit_ + 0.0137 ACCoA_cyt_ → 1 C-mol Protein + 0.004 GA3P + 0.083 NADH + 0.0423 NADP^+^ + 1.21 ADP + 1.21 Pi + 0.0448 CO_2_

X-33/ROL Strain

1.(B) 0.142 Pyr + 0.082 NAD^+^ + 0.0423 NADPH + 1.17 ATP + 0.0068 Rib5P + 0.0143 E4P + 0.0419 OAA + 0.0681 KG + 0.0136 ACCoA_cyt_ → 1 C-mol Protein + 0.0029 GA3P +
0.082 NADH + 0.0423 NADP^+^ + 1.175 ADP + 1.20 Pi + 0.0445 CO2

(2) **Carbohydrate synthesis** (Composition derived from [3])

0.113 Glc6P + 0.053 Fru6P + 0.167 ATP → 1 C-mol Carbohydrate + 0.167 ADP

(3) **Lipids synthesis** (derived from the mean lipid composition from [4])

0.002 Glc6P + 0.0055 Pyr + 0.011 GA3P + 0.006 CO_2_ + 0.039 ACCoA_mit_ + 0.441 ACCoA_cyt_ + 0.07 NADH + 0.599 NADPH + 0.42 ATP + 0.065 O_2_ → 1 C-mol Lipid + 0.07 NAD^+^ +
0.599 NADP^+^ + 0.42 ADP + 0.42 Pi

(4) **RNA synthesis** (derived from the RNA composition [5])

0.056 Pyr + 0.1136 CO_2_ + 0.105 Rib5P + 0.104 NAD+ + 0.075 NADPH + 1.1 ATP + 0.0479 OAA → 1 C-mol RNA + 1.1 Pi + 1.1 ADP + 0.075 NADP^+^ + 0.104 NADH

(5) **DNA synthesis** (composition derived from [5] and the DNA polymerization [3]).

0.051 Pyr +0.102 NAD^+^ + 0.273 NADPH + 1.146 ATP+ 0.132 CO_2_ + 0.102 Rib5P +
0.051 OAA → 1 C-mol DNA + 1.146 Pi + 1.146 ADP + 0.102 NADH + 0.273 NADP^+^

S.6.2. Reaction network used for anNET analysis

| **Abbreviation** | **Reaction** |
| --- | --- |
| HXK | Glc_int_ + ATP → Glc6P + ADP |
| PGI | Glc6P ↔ Fru6P |
| PFK | ATP + Fru6P → ADP + FBP + H |
| FB | FBP + H_2_O → Fru6P + Pi |
| FBA | FBP ↔ DHAP + GA3P |
| TPI | DHAP ↔ GA3P |
| GAPDH | GA3P + NAD^+^ + Pi ↔ H + NADH + 13dpg |
| PGK | 13dpg + ADP ↔ 3PG + ATP |
| GPM | 3PG ↔ 2PG |
| ENO | 2PG ↔ H_2_O + Pep |
| PYK | ADP + H + Pep → ATP + Pyr |
| G6PDH | Glc6P + NADP^+^ → 6pgl + H + NADPH |
| 6PGDH | 6pgl + NADP + H_2_O → Rul5p + NADPH + H + CO_2_tot |
| RPI | Rib5P ↔ Rul5P |
| RPE | Rul5P ↔ Xul5P |
| TK(1)+TA | Rib5P + Xul5P ↔ Fru6P + E4P |
| TK(3) | E4P + S7P ↔ Fru6P + Rib5P |
| TA | GA3P + S7P ↔ Fru6P + E4P |
| TK(1) | Rib5P+ Xul5P ↔ GA3P + S7P |
| G3PDH | DHAP + NADH ↔ glyc3P + NAD^+^ |
| PYRCK | Pyr + ATP + CO_2_tot ↔ OAA + ADP + Pi + H_2_O |
| TPP | (2) Glc6P + UTP + H_2_O → Treh + PPi + UDP + Pi |
| TreP | Treh + H_2_O → (2) Glc_int_ |
| PDC | Pyr → acald + CO_2_tot |
| DHAK | DHA + ATP ↔ DHAP + ADP |
| PMI | Fru6P ↔ Man6P |
| CAT | Metoh + (0.5) O_2_ + 2 NAD^+^ → CO_2_tot + 2 NADH |
| MET | Metoh + Xul5P + (0.5) O_2_ → DHA + GA3P_per_ |

S6.3. Reactions and atom transitions network used in ^13^C-MFA, following the notation of [6].

| **Name** | **Reaction** |
| --- | --- |
| feedGlcB: | FullyGlc> Glc_ext_ |
|  | #abcdef > #abcdef |
| feedGlcC: | CGlc > Glc_ext_ |
|  | #abcdef > #abcdef |
| uptGlc: | Glc_ext_ > Glc_int_ |
|  | #abcdef > #abcdef |
| feedMeOHB: | MetohL > Metoh_ext_ |
|  | #a > #a |
| uptMeOH: | Metoh_ext_ > Metoh_int_ |
|  | #a > #a |
| upt1: | Glu_int_ > Glc6P |
|  | #abcdef > #abcdef |
| upt2: | Metoh_int_ > Form |
|  | #a > #a |
| TRE1: | Glc6P > T6P |
|  | #abcdef > #abcdef |
| TRE2: | T6P > Treh |
|  | #abcdef > #abcdef |
| TRE3: | Treh > Glu_int_ |
|  | #abcdef > #abcdef |
| emp1: | Glc6P <> Fru6P |
|  | #abcdef > #abcdef |
| emp2: | Fru6P > FBP |
|  | #abcdef > #abcdef |
| emp2B | FBP → Fru6P |
|  | #abcdef > #abcdef |
| emp3: | FBP <> DHAP + GA3P |
|  | #abcdef > #cba + #def |
| emp4: | DHAP <> GA3P |
|  | #abc > #abc |
| emp5: | GA3P <> PG3 |
|  | #abc > #abc |
| emp6: | PG3 <> PG2 |
|  | #abc > #abc |
| emp7: | PG2 <> Pep |
|  | #abc > # abc |
| emp8: | Pep > Pyr |
|  | #abc > #abc |
| emp9: | Pyr > ACCoA_cyt_ + CO_2_ |
|  | #abc > #bc + #a |
| emp10: | Pyr + CO_2_ > OAA_mit_ |
|  | #abc + #d > #abcd |
| emp11: | Pyr > Pyrt |
|  | #abc > #abc |
| **Name** | **Reaction** |
| emp11A: | Pyr > Pyr_mit_ |
|  | #abc > #abc |
| emp11B: | Pyr_mit_ > Pyrt |
|  | #abc > #abc |
| emp11C: | NPyr > Pyr_mit_ |
|  | #ABC > #ABC |
| emp11D: | Pyr_mit_ > Pyr_ext_ |
|  | #ABC > #ABC |
| emp12: | ACCoA_cyt_ > ACCoA_mit_ |
|  | #AB > #AB |
| ppp1: | Glc6P > CO_2_ + Rul5P |
|  | #abcdef > #a + #bcdef |
| ppp2: | Rul5P <> Xul5P |
|  | #abcde > #abcde |
| ppp3: | Rul5P <> Rib5P |
|  | #abcde > #abcde |
| ppp4: | Xul5P + E4P <> GA3P + Fru6P |
|  | #ABCDE + #abcd > #CDE + #ABabcd |
| ppp5: | Xul5P + Rib5P <> Sed7P + GA3P |
|  | #abcde + #ABCDE > #ABabcde + #CDE |
| ppp6: | GA3P + Sed7P <> E4P + Fru6P |
|  | #ABC + #abcdefg > #defg + #abcABC |
| TCA1: | Pyr_mit_ > ACCoA_mit_ + CO_2_ |
|  | #ABC > #BC + #A |
| TCA2: | ACCoA_mit_ + OAA > CIT_mit_ |
|  | #AB + #abcd > #dcbaBA |
| TCA3: | CIT_mit_ > αKG + CO_2_ |
|  | #ABCDEF > #ABCEF + #D |
| TCA4: | αKG > SUCC + CO_2_ |
|  | #ABCDE > #BCDE + #A |
| TCA4B: | αKG > SUCC + CO_2_ |
|  | #ABCDE > #EDCB + #A |
| TCA5: | SUCC <> FUM |
|  | #ABCD > #ABCD |
| TCA5B: | SUCC <> FUM |
|  | #ABCD > #DCBA |
| TCA6: | FUM <> MAL |
|  | #ABCD > #ABCD |
| TCA7: | MAL <> OAA |
|  | #ABCD > #ABCD |
| TCA8: | Asp > FUM |
|  | #ABCD > #ABCD |
| Met1: | Form > CO_2_ |
|  | #A > #A |
| Met2: | Xul5P + Form > DHA + GA3P_per_ |
|  | #ABCDE + #F > #FAB + #CDE |
| **Name** | **Reaction** |
| Met2B: | Xul5P + Form > DHA + GA3P_per_ |
|  | #ABCDE + #F > #ABF + #CDE |
| Met3: | DHA > DHAP |
|  | #ABC > #ABC |
| Met4: | GA3P_per_ > GA3P |
|  | #ABC > #ABC |
| BIO1: | Glc6P > Glc6P_bio_ |
|  | #ABCDEF > #ABCDEF |
| BIO2: | Fru6P > Fru6P_bio_ |
|  | #ABCDEF > #ABCDEF |
| BIO3: | ACCoA_cyt_ > ACCoA_bio_ |
|  | #BC > #BC |
| BIO4: | OAA > OAA_bio_ |
|  | #ABCD > #ABCD |
| BIO5: | E4P > E4P_bio_ |
|  | #ABCD > #ABCD |
| BIO6: | Rib5P > Rib5P_bio_ |
|  | #ABCDE > #ABCDE |
| BIO7: | GA3P > GA3P_bio_ |
|  | #ABC > #ABC |
| BIO8: | αKG_mit_ > αKG_bio_ |
|  | #ABCDE > #ABCDE |
| BIO9: | Pyrt > Pyr_bio_ |
|  | #ABC > #ABC |
| CO2out1: | CO_2_ > CO_2__ext |
|  | #A > #A |
| aa_ala: | Pyr <> Ala |
|  | #ABC > #ABC |
| aa_glu: | αKG_mit_ <> Glu |
|  | #ABCDE > #ABCDE |
| aa_asp: | OAA <> Asp |
|  | #ABCD > #ABCD |

References

1. Jordà, J.; Jouhten, P.; Cámara, E.; Maaheimo, H.; Albiol, J.; Ferrer, P. Metabolic flux profiling of recombinant protein secreting *Pichia pastoris* growing on glucose:methanol mixtures. *Microb. Cell. Fact.* **2012**, *11*, 57.
2. Klimacek, M.; Krahulec, S.; Sauer, U.; Nidetzky, B. Limitations in xylose-fermenting Saccharomyces cerevisiae, made evident through comprehensive metabolite profiling and thermodynamic analysis. *Appl. Environ. Microbiol.* **2010**, *76*, 7566–7574.
3. Carnicer, M. Systematic Metabolic Analysis of Recombinant *Pichia pastoris* under Different Oxygen Conditions: A Metabolome and Fluxome Based Study. Ph.D. Thesis, Autonomous University of Barcelona, Bellaterra (Cerdanyola del Vallès), Spain, 2012.
4. Carnicer, M.; Baumann, K.; Töplitz, I.; Sanchez-Ferrando, F.; Mattanovich, D.; Ferrer, P.; Albiol, J. Macromolecular and elemental composition analysis and extracellular metabolite balances of *Pichia pastoris* growing at different oxygen levels. *Microb Cell. Fact.* **2009**, *8*, 65.
5. De Schutter, K.; Lin, Y.C.; Tiels, P.; van Hecke, A.; Glinka, S.; Weber-Lehmann, J.; Rouzé, P.; van de Peer, Y.; Callewaert, N. Genome sequence of the recombinant protein production host *Pichia pastoris*. *Nat. Biotechnol.* **2009**, *27*, 561–566.
6. Wiechert, W.; de Graaf, A.A. *In vivo* stationary flux analysis by ^13^C labeling experiments. *Adv. Biochem. Eng. Biotechnol.* **1996**, *54*, 109–154.

© 2014 by the authors; licensee MDPI, Basel, Switzerland. This article is an open access article distributed under the terms and conditions of the Creative Commons Attribution license (http://creativecommons.org/licenses/by/3.0/).
