# Supplementary material for: Quantitative Metabolomics and Instationary 13C-Metabolic Flux Analysis Reveals Impact of Recombinant Protein Production on Trehalose and Energy Metabolism in Pichia pastoris
Source: Metabolites. 2014 May 5;4(2):281–99. doi: 10.3390/metabo4020281 (PMC4101507; doi:10.3390/metabo4020281)
Supplement: Supplementary File 1 — Supplementary Materials (ZIP, 2359 KB) [file metabolites-04-00281-s001.zip › metabolites-04-00281-supplementary/forMerrin.htm]

S


Type

Title

Amy Ding

\*   
Author to whom correspondence should be addressed. E-Mail: amy.ding@mdpi.com

Received:
1 July 2011 / Accepted: 2 July 2011 / Published: 3 July 2011

**Abstract:**
Test

**Keywords:**
Test

1. Test

test

References

1.         
Jordà, J.; Jouhten, P.; Cámara, E.; Maaheimo, H.; Albiol, J.;
Ferrer, P. Metabolic flux profiling of recombinant protein secreting Pichia
pastoris growing on glucose:methanol mixtures. *Microb. Cell. Fact.* **2012**,
*11*, 57.

2.         
Klimacek, M.; Krahulec, S.; Sauer, U.;
Nidetzky, B. Limitations in xylose-fermenting Saccharomyces cerevisiae,
made evident through comprehensive metabolite profiling and thermodynamic
analysis. *Appl. Environ. Microbiol.* **2010**, *76*, 7566–7574.

3.         
Carnicer, M. Systematic Metabolic Analysis of Recombinant Pichia
pastoris under Different Oxygen Conditions: A Metabolome and Fluxome Based
Study. Ph.D. Thesis, Autonomous University of Barcelona,
Bellaterra (Cerdanyola del Vallès), Spain, 2012.

4.         
Carnicer, M.; Baumann, K.; Töplitz, I.;
Sanchez-Ferrando, F.; Mattanovich, D.; Ferrer, P.; Albiol, J.
Macromolecular and elemental composition analysis and extracellular metabolite
balances of Pichia pastoris growing at different oxygen levels. *Microb Cell.
Fact.* **2009**, *8*, 65.

5.         
De Schutter, K.; Lin, Y.C.; Tiels, P.; van Hecke, A.; Glinka, S.;
Weber-Lehmann, J.; Rouzé, P.; van de Peer, Y.; Callewaert, N. Genome sequence
of the recombinant protein production host Pichia pastoris. *Nat. Biotechnol.*
**2009**, *27*, 561–566.

6.         
Wiechert, W.; de Graaf, A.A. *In vivo* stationary flux analysis by 13C
labeling experiments. *Adv. Biochem. Eng. Biotechnol.* **1996**, *54*,
109–154.
