# Supplementary figures and images for: Quantitative Metabolomics and Instationary 13C-Metabolic Flux Analysis Reveals Impact of Recombinant Protein Production on Trehalose and Energy Metabolism in Pichia pastoris
Source: Metabolites. 2014 May 5;4(2):281–99. doi: 10.3390/metabo4020281 (PMC4101507; doi:10.3390/metabo4020281)

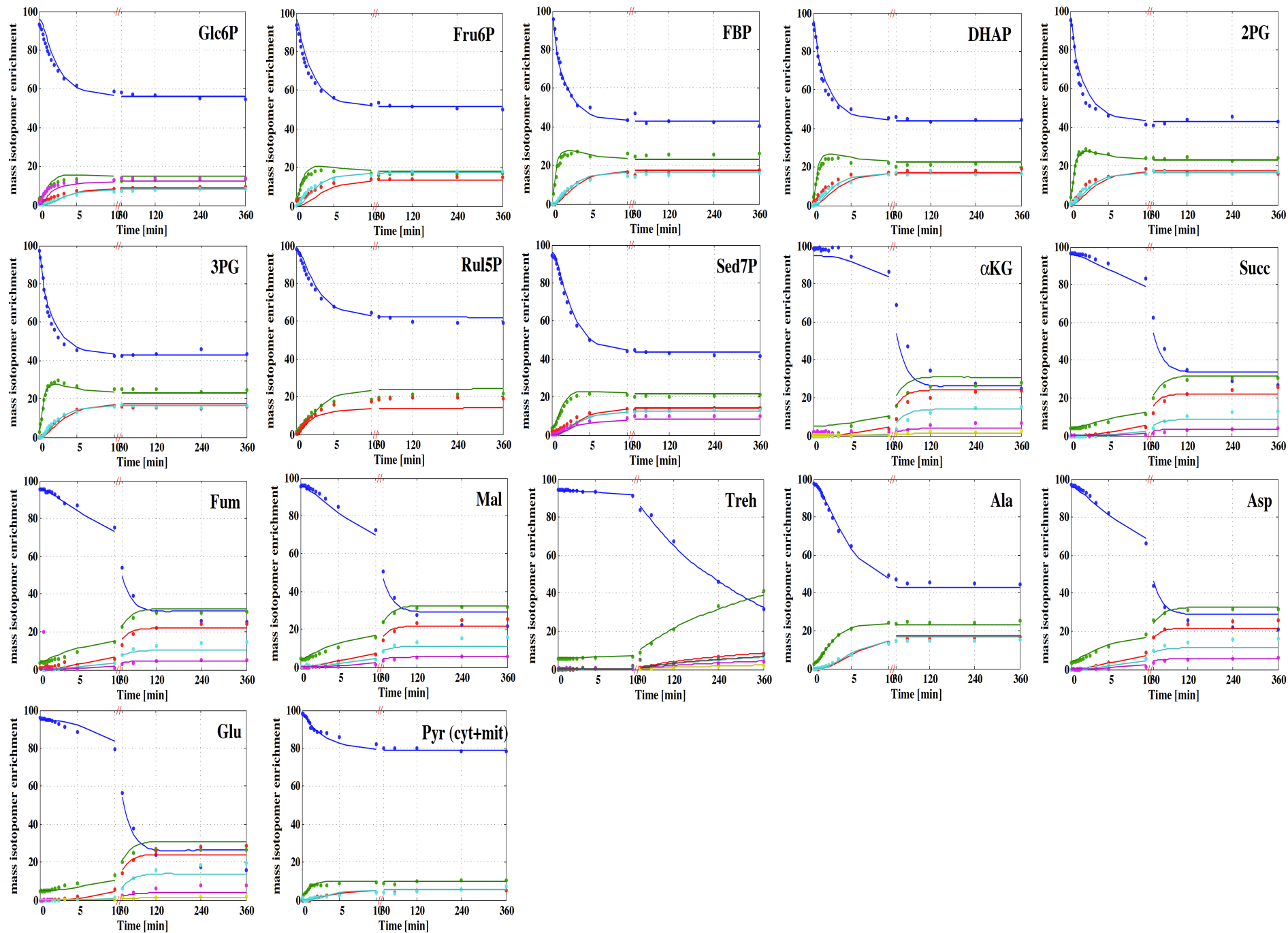

Supplement: Supplementary File 1 — Supplementary Materials (ZIP, 2359 KB) [file metabolites-04-00281-s001.zip › metabolites-04-00281-supplementary/Supplementary file 1_WT.pdf]

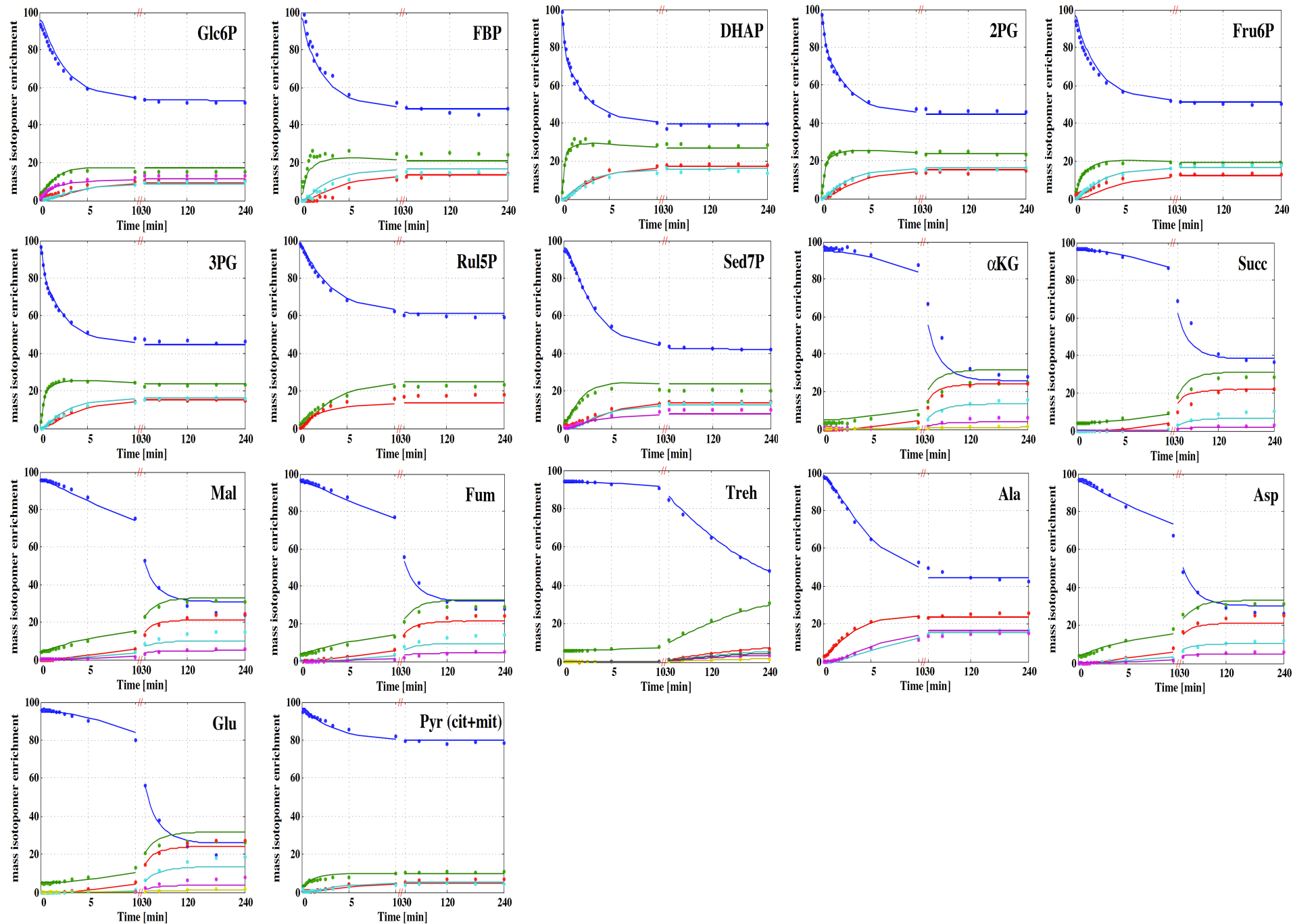

Supplement: Supplementary File 1 — Supplementary Materials (ZIP, 2359 KB) [file metabolites-04-00281-s001.zip › metabolites-04-00281-supplementary/Supplementary file 2_SC.pdf]

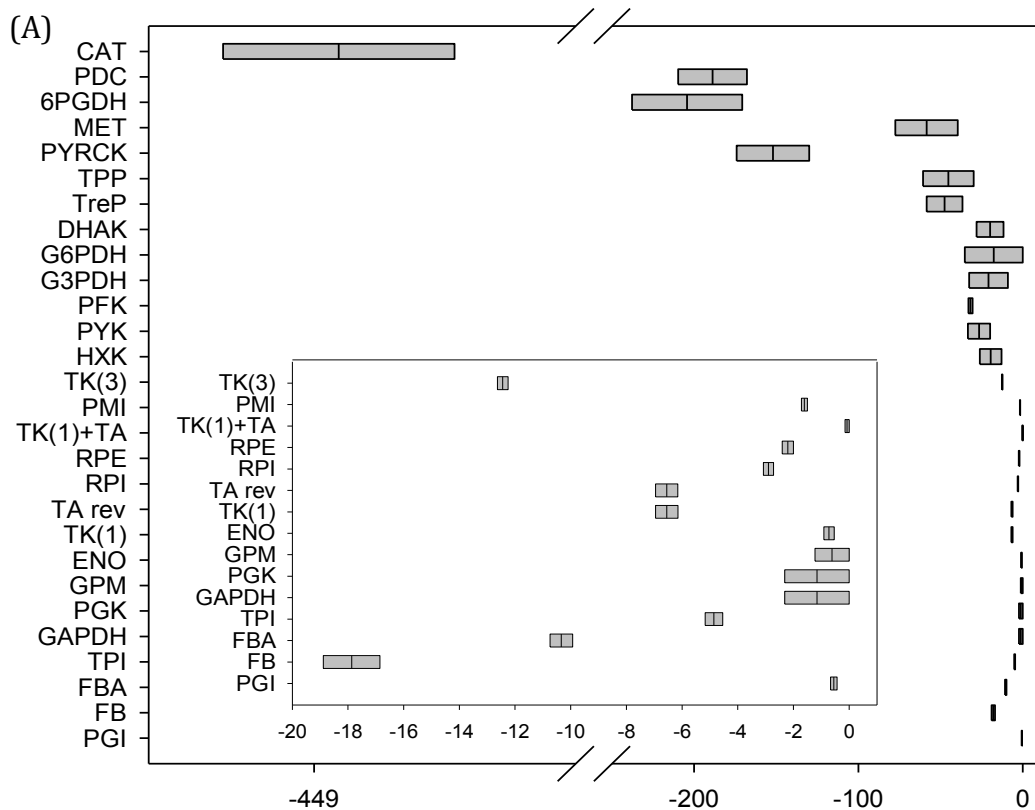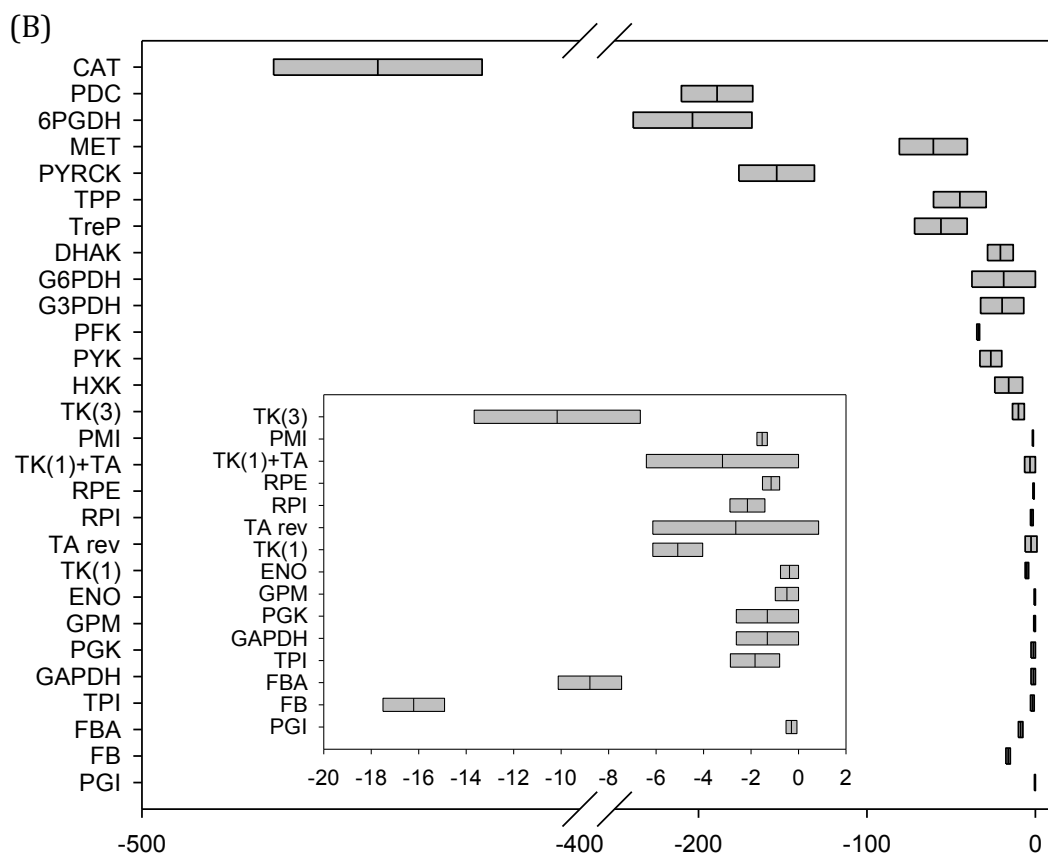

Supplement: Supplementary File 1 — Supplementary Materials (ZIP, 2359 KB) [file metabolites-04-00281-s001.zip › metabolites-04-00281-supplementary/Supplementary file 7.pdf]

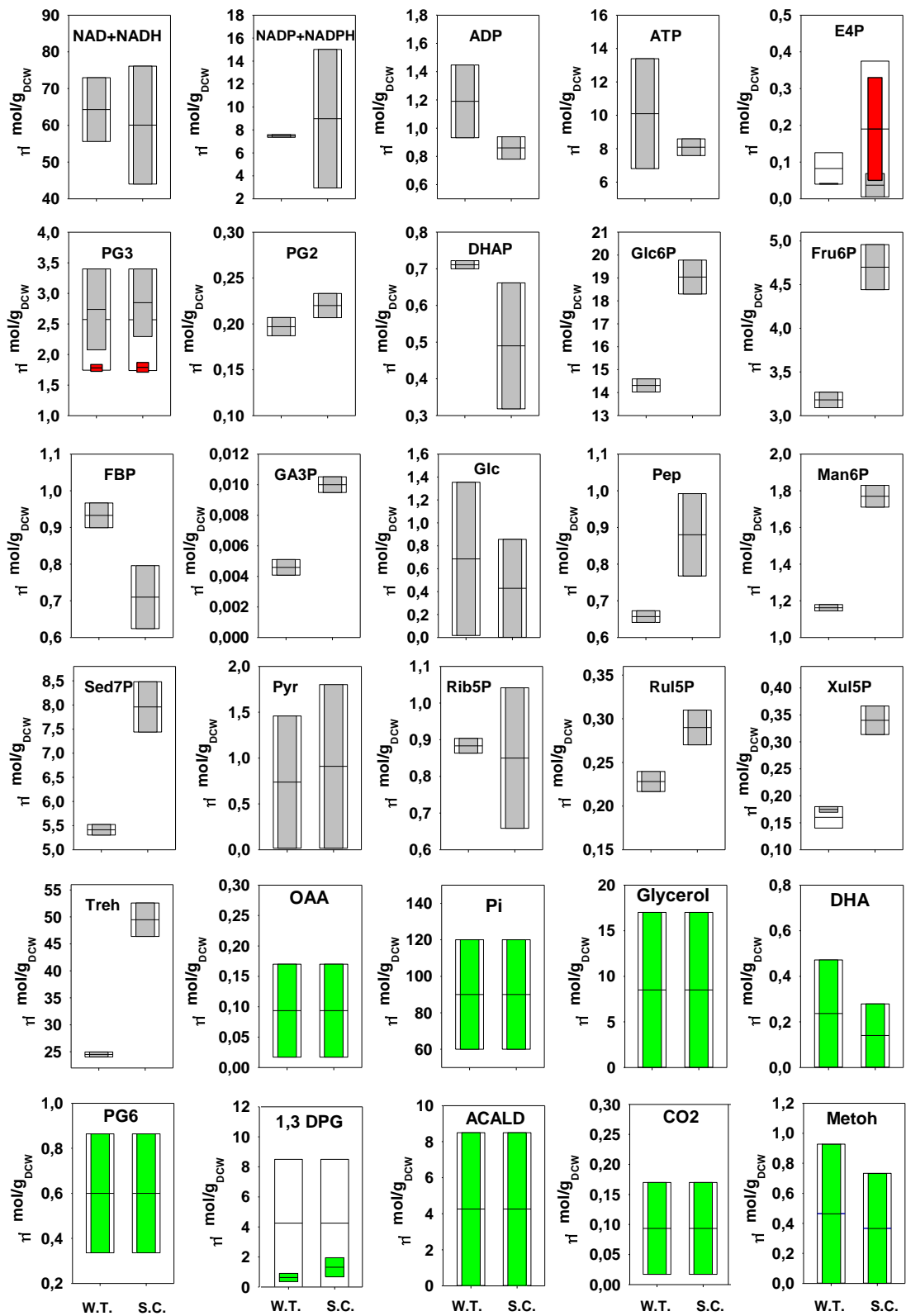

Supplement: Supplementary File 1 — Supplementary Materials (ZIP, 2359 KB) [file metabolites-04-00281-s001.zip › metabolites-04-00281-supplementary/Supplementary file 8.pdf]

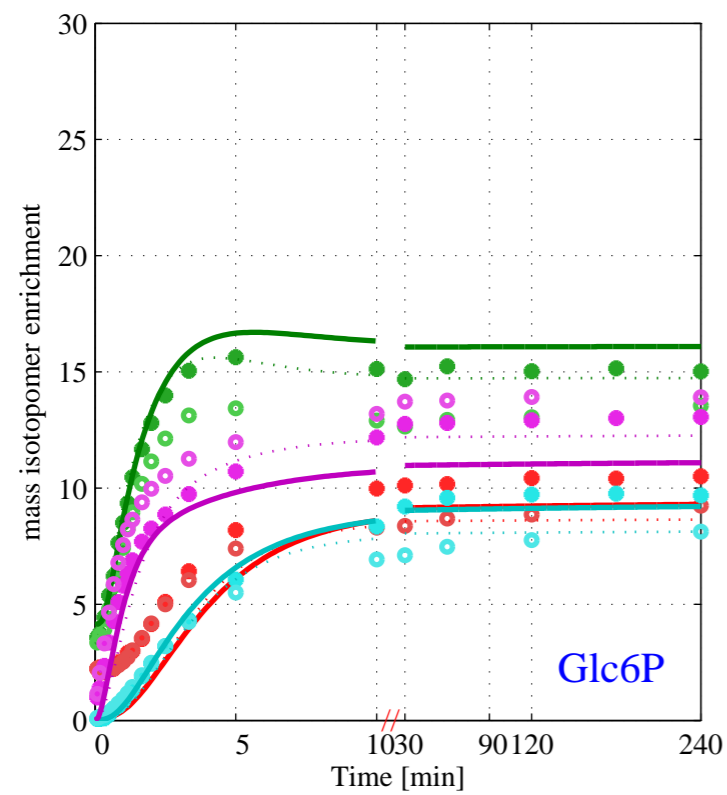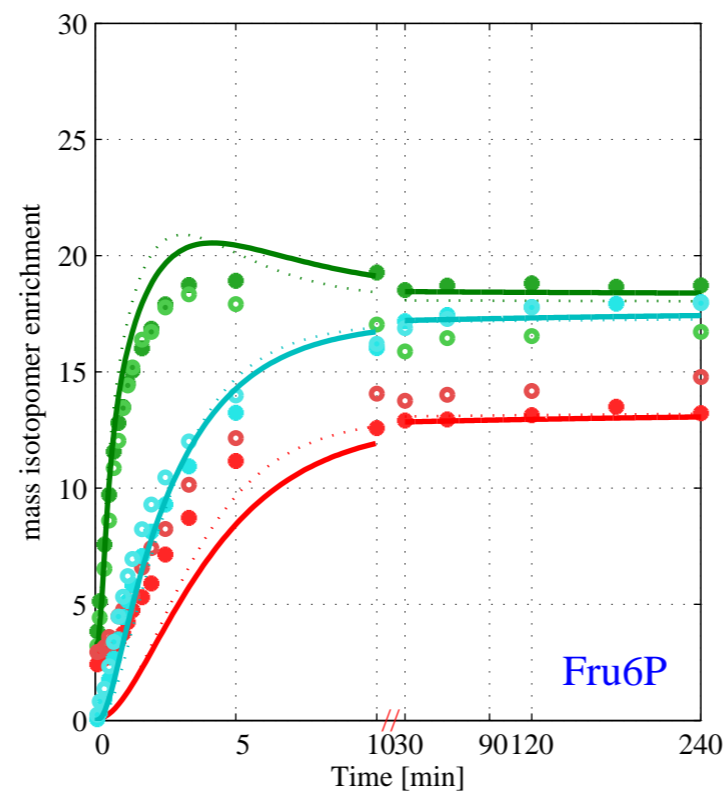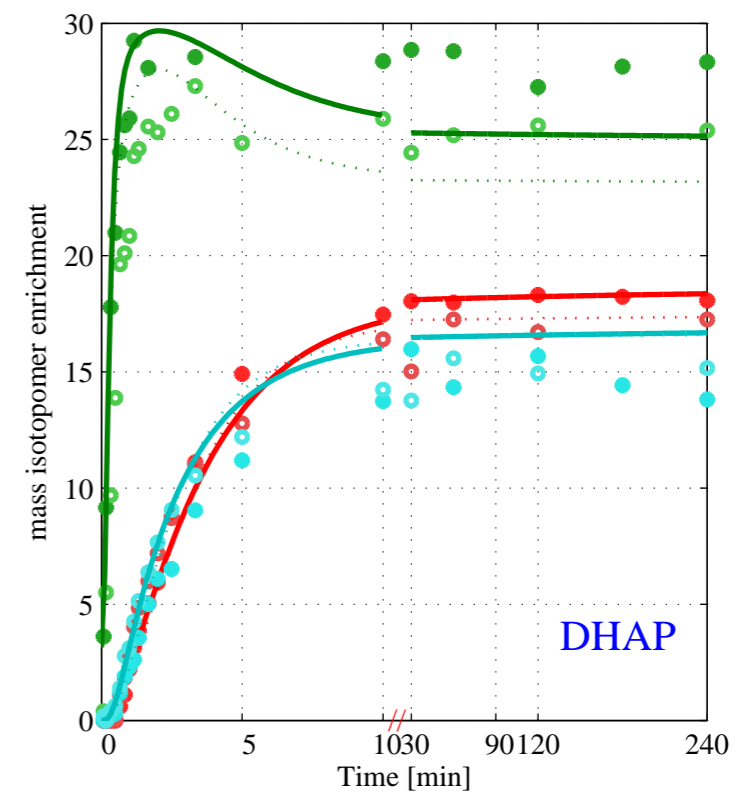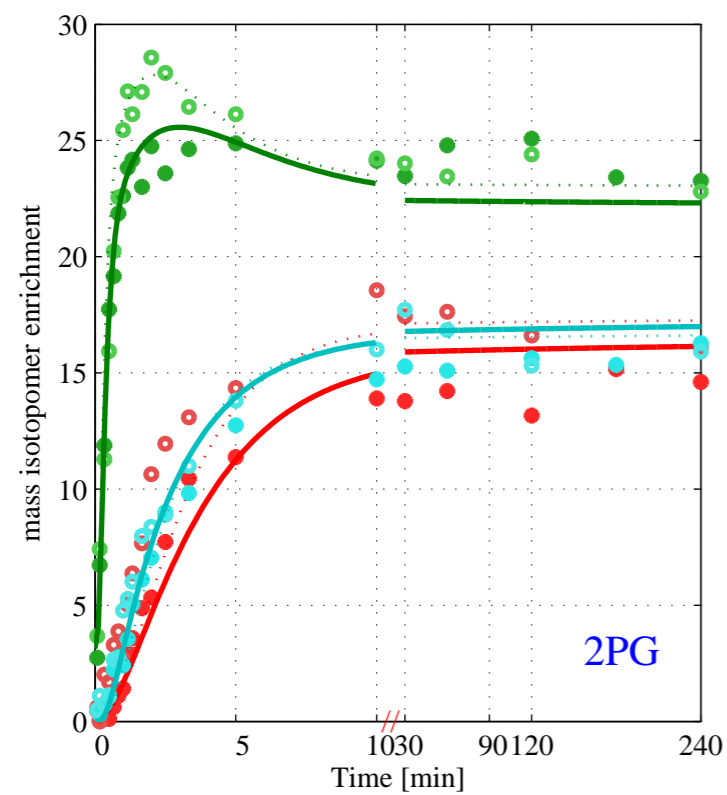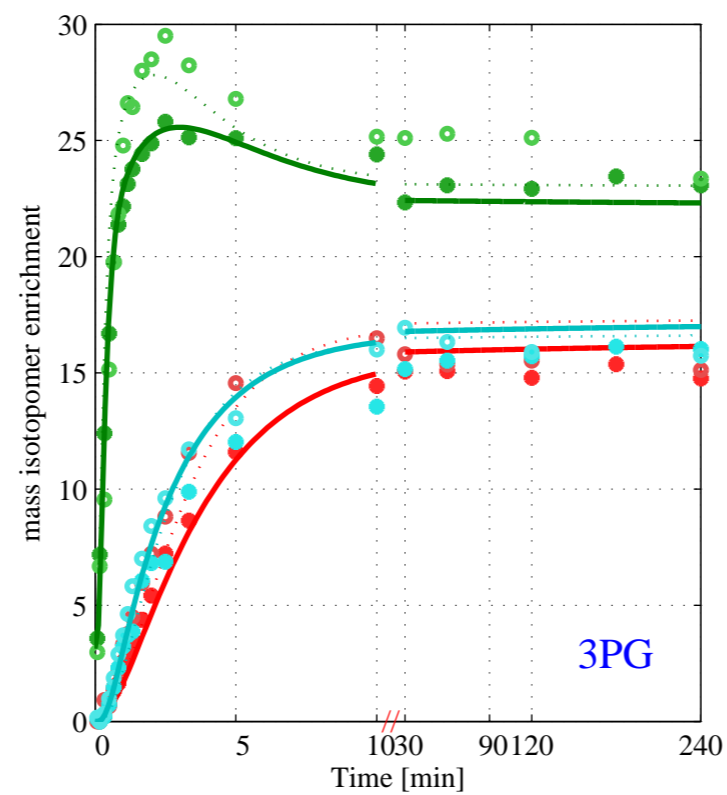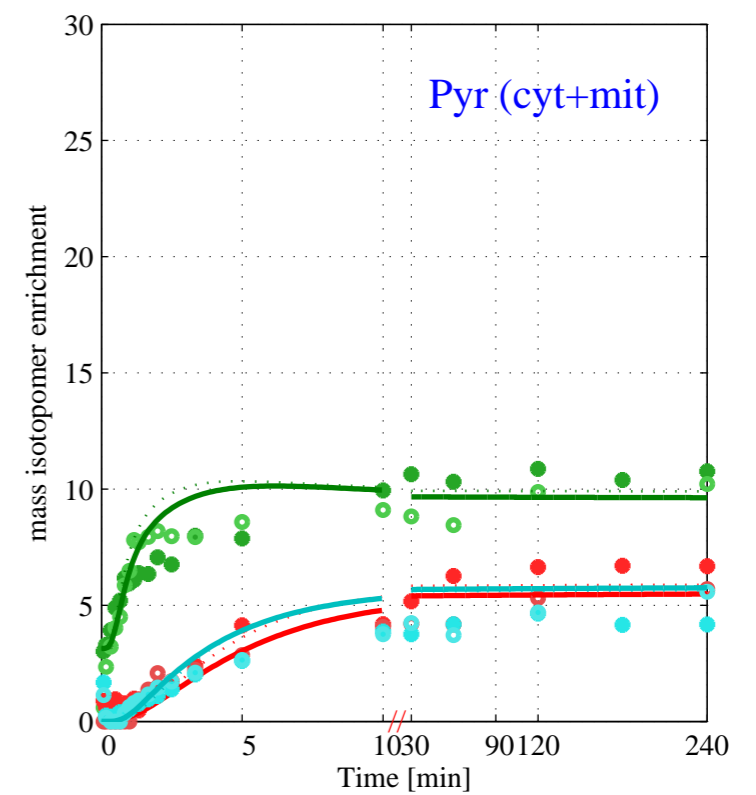

Supplement: Supplementary File 1 — Supplementary Materials (ZIP, 2359 KB) [file metabolites-04-00281-s001.zip › metabolites-04-00281-supplementary/Supplementary file 9.pdf]
